# High-salt diet induces immune-independent re-differentiation, metabolic shut down and cell cycle arrest of melanoma

**DOI:** 10.1038/s41419-025-08329-x

**Published:** 2025-12-20

**Authors:** Clivia Lisowski, Natascha E. Stumpf, Katarzyna Jobin, Daniela Klaus, Melanie Eichler, Ann-Kathrin Baumgart, Olena Babyak, Mirjam Meißner, Agnes Schröder, Patrick Neubert, Vanessa Schmitt, Marcel Michla, Süleyman Bozkurt, Jelena Zurkovic, Daniel Hinze, Jana Liebing, Matthias Eckhardt, Christoph Heuser, Lea Seep, Annkristin Heine, Janine Becker-Gotot, Christoph Thiele, Christian Münch, Thomas Becker, Jonathan Jantsch, Christoph Wilhelm, Michael Hölzel, Christian Kurts

**Affiliations:** 1 Institute for Molecular Medicine and Experimental Immunology, University of Bonn, Bonn, Germany; 2 Department of Pediatric Immunology and Rheumatology, University Hospital Bonn, Bonn, Germany; 3 Department of Microbiology, Biocenter, University of Würzburg & Institute of Systems Immunology, Würzburg, Germany; 4 Department of Orthodontics, University Hospital Regensburg, Regensburg, Germany; 5 Institute for Microbiology and Hygiene, University of Regensburg, Regensburg, Germany; 6 Institute of Clinical Chemistry & Clinical Pharmacology, University Hospital Bonn, Bonn, Germany; 7 Baker Heart and Diabetes Institute, Melbourne, VIC Australia; 8 Institute of Molecular Systems Medicine, Goethe University, Frankfurt am Main, Germany; 9 Biochemistry & Cell Biology of Lipids, Life & Medical Sciences (LIMES)-Institute, University of Bonn, Bonn, Germany; 10 Institute of Experimental Oncology, University Hospital Bonn, Bonn, Germany; 11 Institute for Biochemistry and Molecular Biology, University of Bonn, Bonn, Germany; 12 Computational Biology, Life & Medical Sciences (LIMES) Institute & Bonn Center for Mathematical Life Sciences, University of Bonn, Bonn, Germany; 13 Institute of Experimental Immunology & Medical Clinic III, University Hospital Bonn, Bonn, Germany; 14 Institute for Medical Microbiology, Immunology and Hygiene, Center for Molecular Medicine Cologne (CMMC), University Hospital Cologne & Faculty of Medicine, University of Cologne, Köln, Germany; 15 Present Address: Division of Functional Immune Cell Modulation, Leibniz Institute for Immunotherapy, Regensburg, Germany & Internal Medicine III—Haematology and Oncology, University Hospital Bonn, Bonn, Germany

**Keywords:** Melanoma, Cancer

## Abstract

High salt diet (HSD) is known to reduce cancer growth in some tumor models, which has been attributed to tissue accumulation of sodium that enhances local anti-tumor immunity. Here, we show that a HSD inhibits melanoma growth independent of sodium accumulation and immune cells in skin and lung. Melanoma cells from mice on a HSD upregulated the metabolic inhibitor Tuberous sclerosis complex 2 (TSC2), causing metabolic shutdown despite nutrient availability. Furthermore, Microphthalmia-associated transcription factor (MITF), a crucial regulator of melanoma metabolism and differentiation, was upregulated, resulting in enhanced melanogenesis and cell cycle arrest. Thus, a HSD reversed the de-differentiation of melanoma cells and promoted their re-differentiation into a “normal” melanocytic state. These findings suggest that the anti-tumor effect of HSD may be tumor-specific and in some cases immune cell-independent.

## Introduction

Cutaneous melanoma remains one of the most aggressive forms of skin cancer, ranking 17th among all cancers worldwide. It has the highest prevalence in Western Europe and North America, with approximately 330,000 recorded cases and 58,000 deaths globally in 2022 [1]. Malignant melanoma originates from melanocytes, specialized pigment-producing cells in the skin that protect keratinocytes from UV-induced DNA damage [2]. During embryonic development, melanocytes arise from melanoblasts in the neural crest via coordinated processes of migration, proliferation, and differentiation [3]. This is regulated by various transcription factors, such as SOX10 (sex-determining region Y-box 10), SCF/c-KIT (Stem cell factor), and MITF (Microphthalmia-associated transcription factor), the master regulator of melanocyte development and differentiation [4–6]. Notably, differentiation is not a one-way process but depends on the balance and expression of several transcriptional networks in a highly dynamic manner [7]. A hallmark of melanoma, as with other cancers, is cellular de-differentiation characterized by high phenotypic plasticity or “phenotype switching”, leading to a heterogeneous tumor microenvironment (TME) [8]. In melanoma, markers from early development re-appear, while those indicative of a differentiated state disappear [7, 9]. MITF is particularly important, as it was shown that melanoma cells exhibit different states based on its expression levels. The rheostat model suggests that melanomas can be either slow-cycling, invasive and dedifferentiated, or highly proliferative and less invasive, or terminally differentiated and non-proliferative [10]. Another hallmark of cancers is an aberrant cellular metabolism that provides necessary energy for rapid and uncontrolled proliferation. Melanoma, in particular, was shown to consume large amounts of glucose that is converted into lactate via aerobic glycolysis (Warburg effect) [11]. Despite their dependence on glycolysis, melanoma cells maintain mitochondrial activity and oxidative phosphorylation, allowing them to adapt to various environmental conditions, which contributes to invasion, metastasis, and therapy resistance [12, 13]. This metabolic flexibility increases the heterogeneity of melanoma, with various stages of differentiation and metabolism interconnected at transcriptional and translational levels, even though the nature of these networks is not fully elucidated [14–16]. In addition, MITF regulates genes essential for energy production and organelle biosynthesis and function [5, 10]. This positions MITF as a central cellular hub that links differentiation and metabolism. Western dietary habits are characterized by a high intake of sugar, fat, and salt [17]. The effects of high salt consumption have been investigated in the context of cardiovascular diseases, autoimmunity, and cancer [18]. It has been shown that salt can both promote and inhibit tumor growth and metastasis, even in the same cancer entity [19–21]. These effects are largely attributed to pro- or anti-inflammatory responses of immune cells like CD8+ T cells [22], Th17 cells [23], or Myeloid-derived suppressor cells (MDSCs) [24]. In case of melanoma, a high salt diet was described to affect the interaction of NK cells with the microbiota [25] and the function of MDSCs [26]. However, whether a HSD directly affects tumor cells remains unknown. Here, we aimed to clarify this and found that a HSD induces re-differentiation of melanoma cells, resulting in metabolic shutdown and cell cycle arrest in an immune cell-independent manner, ultimately leading to reduced tumor growth.

## Results

**Fig. 1:**
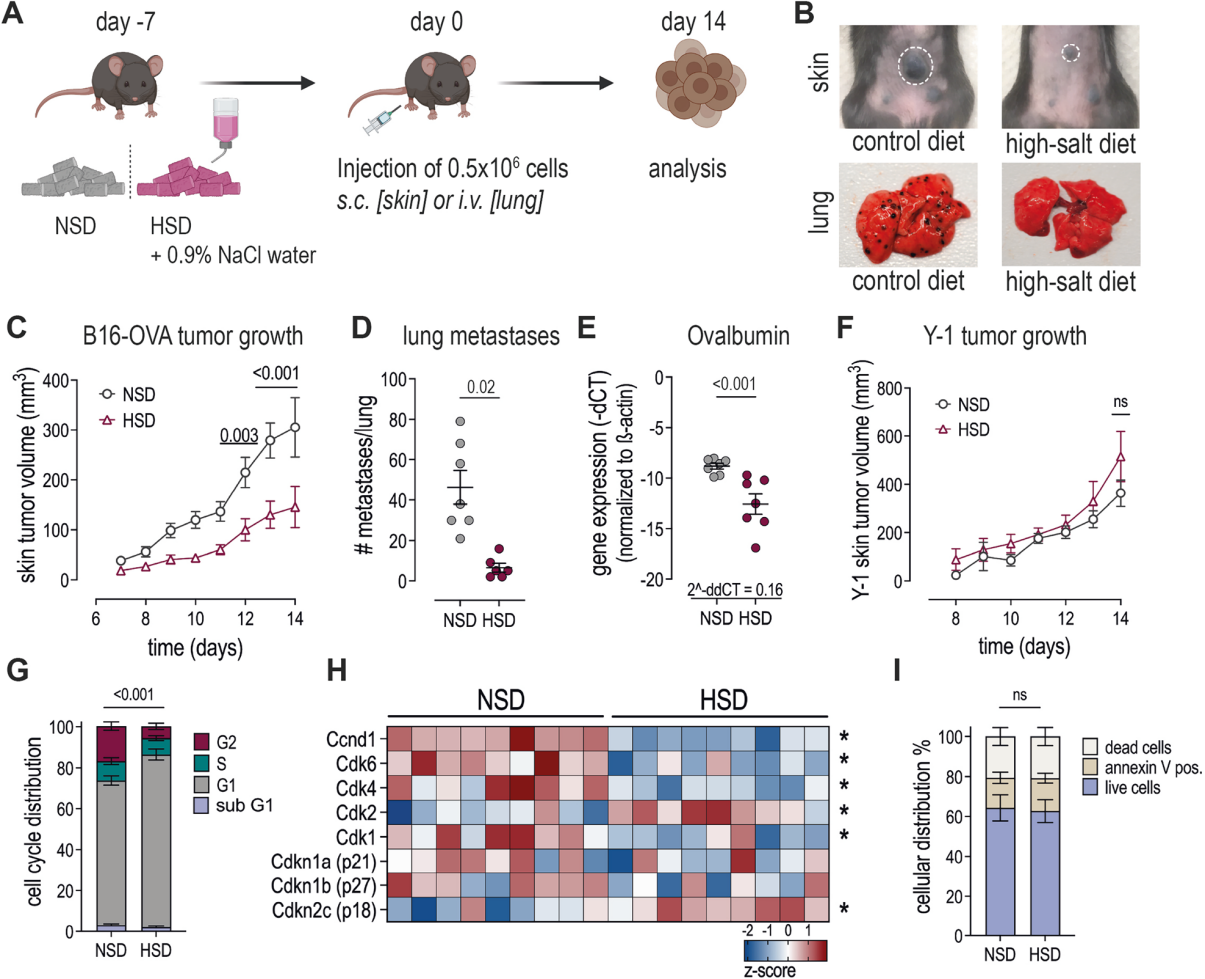
HSD reduces B16-OVA melanoma growth in skin and lung, but does not inhibit growth of Y-1 adrenal cancer cells. **A** Experimental set-up. C57BL/6 mice were fed a NSD or HSD for 7 days before injection of B16-OVA cells. Mice were kept on the diets for 14 days while tumor growth was assessed. **B** Representative images of skin tumors (upper panel) and lung metastasis (lower panel) of B16-OVA melanoma from two independent experiments. **C** Tumor volume of B16-OVA skin melanoma over the course of 14 days. n = ≥ 5 independent experiments with 5–10 mice/group each. 2-way-ANOVA, with Sidaks multiple comparison test. **D** Number of B16-OVA lung metastasis (manually counted). n = 2 independent experiments with 3–4 mice/group each. Linear mixed effect model with treatment (HSD vs. NSD) as fixed factor and experimental replicate as random factor. Data are presented as mean ± SEM. **E** Gene expression level Ovalbumin in whole lung tissue as readout for presence of cancer cells. n = 2 independent experiments with 3–4 mice/group each. Linear mixed effect model with treatment (HSD vs. NSD) as fixed factor and experimental replicate as random factor. Data are presented as mean ± SEM. **F** Volume of skin Y-1 tumors over the course of 14 days. n = 2 independent experiments with 5–10 mice/group each. Two-way ANOVA mixed-effect analysis comparing row means with Sidak’s multiple comparison test. **G** Cell cycle distribution skin B16-OVA cells ex vivo. n = 2 independent experiments with ≤10 mice/group each. Linear mixed effect model with treatment (HSD vs. NSD) as fixed factor and experimental replicate as random factor. Data are presented as mean ± SEM. **H** Protein level (z-score) of cell cycle regulators (mass spectrometry of B16-OVA skin tumor cells ex vivo). Two-way ANOVA mixed-effect analysis with uncorrected Fishers LSD test. Stars indicates significance *p* ≤ 0.01. **I** Apoptosis assay of B16-OVA skin tumor cells ex vivo. n = 1 experiment with 5 mice/group. Unpaired two-tailed t-test. Data are presented as mean ± SEM. All analyses were done after sacrificing the mice on day 14 after tumor injection.

A high salt diet has been reported to invigorate the immune defense against cutaneous leishmaniasis [27]. Hence, we investigated whether the diet can strengthen the immune defense against melanoma as well. We exposed C57BL/6J mice to a normal salt diet (NSD) or a high salt diet (HSD) for one week before s.c. injection of B16-OVA cells and followed tumor growth for 14 days while maintaining the respective diets (Fig. 1A). We observed a significant reduction in skin tumor volume under HSD, indicating reduced or delayed proliferation of tumor cells (Fig. 1B, C), while the overall body weight did not differ between NSD and HSD groups (Supplementary Fig. 8A). To test whether this effect was restricted to the skin or exerted systemically, we injected B16-OVA cells i.v. and analyzed the number of metastases as well as the expression of OVA in the lung after 14 days. We counted almost 8-fold less metastases (Fig.1B, D) and OVA expression, which we used as a readout for the presence of melanoma cells, was more than 6-fold reduced (Fig. 1E), indicating that the growth inhibitory effect of HSD was not restricted to the skin, but acted systemically. To determine if the observed growth reduction was a general effect or specific to melanoma, we subcutaneously injected Y-1 cells, an adrenal gland cancer cell line, and monitored tumor growth over 14 days. The growth of Y-1 tumors was not inhibited by HSD, suggesting that the inhibitory effect of HSD was specific to melanoma (Fig. 1F). To investigate whether the reduced tumor growth under HSD was due to growth arrest or increased cell death, we analyzed the frequency of cells in different cell cycle phases and used annexin V and a cell viability staining to assess the number of apoptotic tumor cells ex vivo. HSD significantly increased the frequency of cells in G1/G0 phase, suggesting cell cycle arrest (Fig. 1G). This finding was corroborated by mass spectrometric analysis of tumor cells, which revealed a significant downregulation of CyclinD1, CDK4, and CDK6, key regulators of G1-S transition, in HSD tumors. This inhibition is likely due to a significant increase in p18, a cell cycle inhibitory protein that targets CDK4/6 (Fig. 1H). Furthermore, annexin V and cell viability staining showed no increase in annexin positive or dead cells, indicating that HSD induces growth arrest rather than cell death (Fig. 1I). We hypothesized that sodium accumulation in the tumor, surrounding skin tissue, or lungs might contribute to this growth defect, which prompted us to measure the ion concentration in these tissues. Surprisingly, we found no differences in sodium, potassium, chloride, or calcium levels (Supplementary Fig. 1A–C). To verify our findings, we assessed the expression levels of NFAT5 and SGK1, which are sensors of sodium and osmotic stress, respectively. Consistent with the observed lack of salt accumulation in the tissue, HSD did not increase the expression levels of either sensor (Supplementary Fig. 1D, E). Finally, to confirm that the reduced tumor growth was not due to salt or osmotic stress sensing via SGK1 or NFAT5, we generated B16-OVA cells deficient for either SGK1 or NFAT5 (Supplementary Fig. 1H and Supplementary Figure 6) and injected them subcutaneously into mice on NSD or HSD. The tumors of HSD mice still exhibited reduced growth (Supplementary Fig. 1F, G), indicating that the inhibitory effect of HSD on in vivo melanoma growth was not mediated by direct effects of sodium or osmotic stress on the tumor cells.

**Fig. 2:**
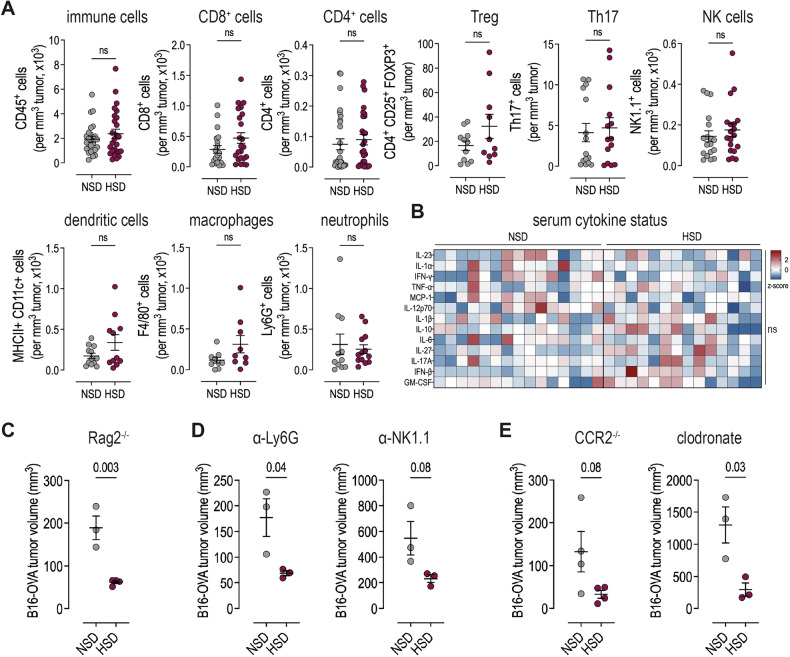
HSD does not invigorate with the anti-tumor immune response. **A** Immune cell count per mm^3^ B16-OVA skin tumor on day 14 after injection, determined by flow cytomtery. n = 3 independent experiments with 2–4 mice/group each. Linear mixed effect model with treatment (HSD vs. NSD) as fixed factor and experimental replicate as random factor. Data are presented as mean ± SEM. **B** Serum cytokine profile on day 14; n = 3 independent experiments with 3–9 mice/group each. Multiple unpaired t-tests without correction for multiple comparisons. **C** Tumor volume of B16-OVA skin melanoma on day 14 in RAG2^−/−^ mice. **D** Tumor volume of B16-OVA skin melanoma on day 14 in wild-type mice treated with anti-Ly6G or anti-NK1.1 antibodies. **E** Tumor volume of B16-OVA skin melanoma on day 14 in CCR2^−/−^ mice or wild-type mice treated with clodronate. **C**–**E** n = 1 experiment with 3–4 mice/group each. Unpaired two-tailed t-test. Data are presented as mean ± SEM.

A high salt diet has been shown to stimulate various immune cells due to salt accumulation within specific tissues [18, 28]. However, since our model showed no sodium accumulation, we asked whether the growth defect could still be attributed to an altered immune response. To investigate this, we quantified the tumor-infiltrating immune cell populations by flow cytometry. As anticipated, neither the overall number nor distribution of CD45+ cells nor the number of CD8+, CD4+, TH17+ or Treg-cells as well as macrophages, dendritic cells, neutrophils or NK cells per mm3 of tumor was changed between NSD and HSD tumors (Fig. 2A and Supplementary Fig. 7A, E). Additionally, we measured cytokine concentrations in serum of tumor-bearing mice. We did not observe any differences in serum cytokine levels (Fig. 2B), indicating that HSD did not have pro-inflammatory effects. Finally, we confirmed this on a functional level by feeding RAG2-/- mice that lack B-and T-cells a HSD and assed B16-OVA tumor growth. However, these mice still exhibited suppressed tumor growth during HSD, arguing against a role of the adaptive immune system in the observed phenotype (Fig. 2C and Supplementary Fig. 7F). We also depleted neutrophils with an anti-Ly6G antibody and NK cells with an anti-NK1.1 antibody, but neither treatment prevented the tumor-suppressive effect of the HSD (Fig. 2D and Supplementary Fig. 7C, D). To investigate the role of macrophages in HSD-mediated tumor suppression we (i) used CCR2-/- mice, which cannot release inflammatory monocytes from the bone marrow and therefore show impaired recruitment to the tumor, and (ii) treated mice with clodronate liposomes, which deplete phagocytic cells. However, both treatments had no effect on the tumor suppression induced by HSD (Fig. 2E). In addition, we analyzed whether depletion of MDSCs with gemcitabine and 5-fluorouracil, which are known to specifically target MDSCs [29, 30], would resume tumor growth as previously suggested [26]. Both drugs significantly reduced tumor growth in NSD mice, confirming their efficacy, while in HSD mice, MDSC depletion did not lead to resumed tumor growth, and the growth-inhibitory effect of HSD remained unchanged (Supplementary Fig. 7B). This indicates that the effect of HSD in our model is independent of MDSCs. Of note, OVA expression in B16-OVA skin tumors was not altered upon HSD, excluding immunogenic escape as well (Supplementary Fig. 7G). Taken together, we did not detect sodium accumulation or a notable increase in the number of immune cells in the TME of HSD mice. By using KO mouse strains and depletion antibodies, we ruled out enhanced tumor suppression by the innate and adaptive immune system upon HSD. However, we still observed impaired melanoma growth, suggesting an additional mechanism by which HSD affects melanoma cells.

**Fig. 3:**
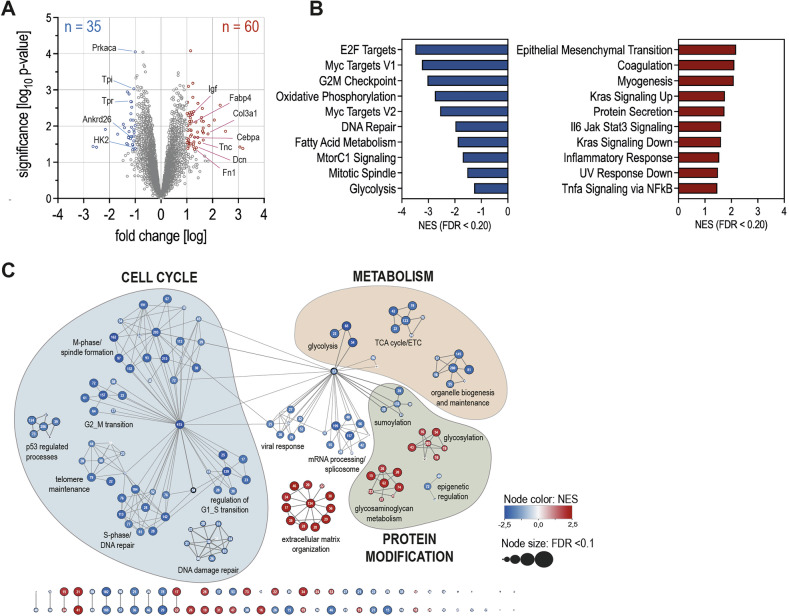
HSD downregulates cell cycle and cellular metabolism on a transcriptional level. Bulk RNA sequencing (3’ mRNA sequencing on the Illumina HiSeq2500 platform) was performed on B16-OVA skin tumor cells that were harvested from 5 NSD and 5 HSD mice on day 14 after tumor injection. **A** Volcano plot of differentially expressed genes in tumor cells isolated from mice on HSD vs. NSD. **B** Pathway enrichment analysis using GSEA (MsigDB) for DEGs from HSD vs. NSD. Blue bars indicate deceased expression (FDR < 0.2). Red bars indicate increased expression (FDR < 0.2). **C** Reactome analysis using GSEA (MsigDB) for DEGs from HSD vs. NSD. Red circles indicate increased expression. Blue circles indicate decreased expression. White numbers indicate genes included in that particular cluster. Size of circles indicate significance (FDR < 0.1).

To determine differences in tumor tissue from NSD and HSD mice we performed 3´-mRNA sequencing and identified 9667 genes, of which 95 were differentially expressed (DEGs). 35 DEGs were significantly downregulated (LogFC <1) and 60 DEGs were significantly upregulated (LogFC >1) under HSD (Fig. 3A). Global gene set enrichment analysis revealed a slight enrichment of extracellular matrix organization (ECM) as well as epithelial-mesenchymal transition (EMT) pathways, while pathways involved in cell cycle regulation and metabolism were strongly downregulated (Fig. 3B). Reactome network analysis also showed downregulation of gene networks involved in cell cycle regulation and metabolism (Fig. 3C). The increase in ECM genes prompted us to investigate whether HSD-induced ECM changes might affect tumor cell engraftment and delay tumor growth under HSD. The ECM comprises collagens, elastin, adhesive glycoproteins, and proteoglycans. Sirius red staining to quantify collagen bundles within the tumor tissue and surrounding skin revealed a slight increase in the percentage of fibrotic area under HSD (Supplementary Fig. 2A–C). However, mRNA expression of collagens and enzymes involved in ECM as well as EMT did not show any differences (Supplementary Fig. 2D). To clarify on a functional level whether reduced tumor growth was due to impaired tumor engraftment, we switched diets 3 days after tumor cell injection using our lung tumor model, which is sensitive enough to detect slight changes in tumor growth earlier than the skin tumor model. We found that tumors, injected into mice on HSD, which were then switched to NSD 3 days post-injection, exhibited slightly delayed but ultimately enhanced growth compared to the HSD group, indicating that HSD delays proliferation but not engraftment (Supplementary Fig. 2E). Conversely, tumors injected into NSD mice, which were switched to HSD, exhibited significantly reduced growth compared to NSD mice, demonstrating that HSD suppressed tumor growth rather than engraftment. Additionally, we investigated whether the inhibitory effect of HSD was transient or induced long-lasting changes in our skin tumor model. For this we switched diets from HSD to NSD on day 14 and monitored tumor growth for another 14 days. Tumor growth in these mice remained low and only began to increase slightly, but not significantly, on day 28 (Supplementary Fig. 2F), suggesting that the HSD effects require at least 10–12 days to be “washed out,” allowing resumed growth. This implies that a prolonged HSD potentially leads to a complete halt. Due to ethical considerations, we could not extend the experiment duration (especially for the control groups), which would have allowed a longer observation of tumors. Taken together, reactome analysis indicated an upregulation of genes involved in extracellular matrix organization, although we could not further verify these finding, while signaling pathways related to cell cycle regulation and metabolism were strongly downregulated.

**Fig. 4:**
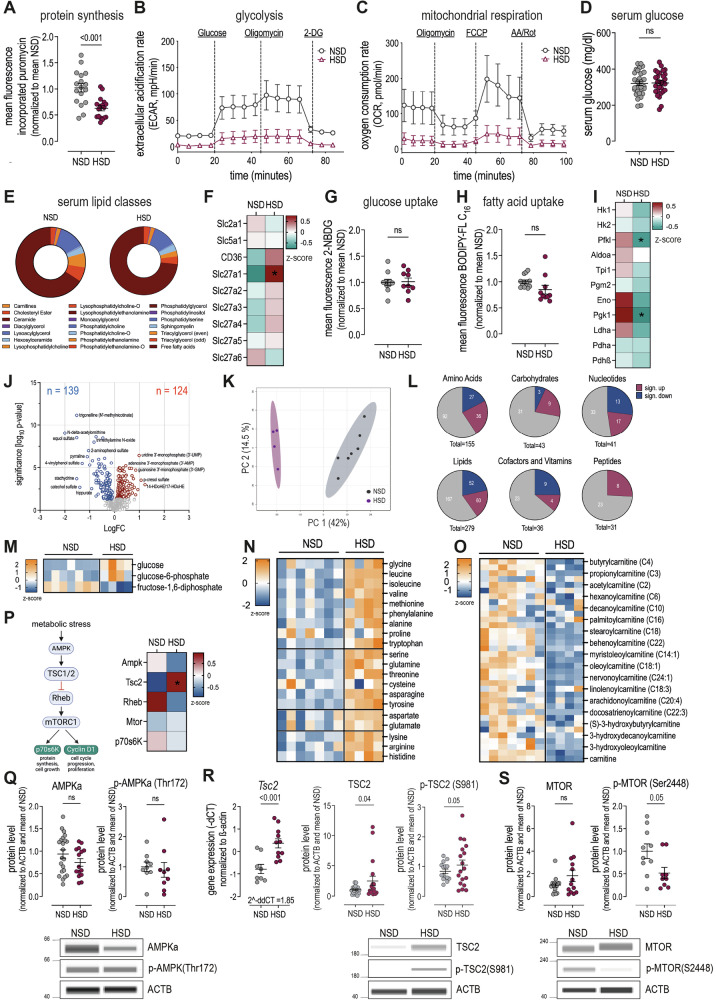
HSD upregulates TSC2 and causes shutdown of cellular metabolism. **A** Geometric mean of fluorescence intensity of puromycin incorporated into B16-OVA tumor cells ex vivo, normalized to mean of the control group. *n* = 3 independent experiments with 3–9 mice/group each. Linear mixed effect model with treatment (HSD vs. NSD) as fixed factor and experimental replicate as random factor. Data are presented as mean ± SEM. **B** Extracellular acidification rate in Seahorse metabolic flux assay of B16-OVA cells ex vivo. n = 2 independent experiments with 5–10 mice/group each. **C** Oxygen consumption rate in Seahorse metabolic flux assay of B16-OVA cells ex vivo. n = 2 independent experiments with 5–10 mice/group each. **D** Serum glucose level on day 14. n = 3 independent experiments with 8–10 mice/group each. Linear mixed effect model with treatment (HSD vs. NSD) as fixed factor and experimental replicate as random factor. Data are presented as mean ± SEM. **E** Serum lipid profile (pmol) on day 14. n = 4 independent experiments with 5–10 mice/group each. **F** Grouped z-score of gene expression level of genes coding for glucose and fatty acid uptake proteins. n = 2-3 independent experiments with 5 mice/group each. Two-way ANOVA mixed-effect analysis with uncorrected Fishers LSD test. **G** Geometric mean of fluorescence intensity of 2-NBDG, a glucose analog, taken up by B16-OVA tumor cells ex vivo, normalized to mean of the control group. n = 2 independent experiments with 2–5 mice/group each. Linear mixed effect model with treatment (HSD vs. NSD) as fixed factor and experimental replicate as random factor. Data are presented as mean ± SEM. **H** Geometric mean of fluorescence intensity of BODIPY-FL-C_16_, taken up by B16-OVA tumor cells ex vivo, normalized to mean of the control group. n = 2 independent experiments with 2–5 mice/group each. Linear mixed effect model with treatment (HSD vs. NSD) as fixed factor and experimental replicate as random factor. Data are presented as mean ± SEM. **I** Grouped z-score of gene expression level of genes coding for enzymes involved in glycolysis. n = 2 independent experiments with 5 mice/group each. Two-way ANOVA mixed-effect analysis with uncorrected Fishers LSD test. **J** Volcano plot of fold change HSD/NSD of tumor metabolites on day 14. n = 1 experiment with 4–7 mice/group. **K** PCA analysis of tumor sample isolates from mice on NSD and HSD. n = 1 experiment with 4–7 mice/group. **L** Analysis of de-regulated metabolites in different nutrient classes. (**M**) Z-score of glucose, glucose-6-phosphate and fructose-1,6-diphosphate in tumor samples. **N** Z-score of intracellular amino acids levels. **O** Z-score of different intracellular carnitine classes. **P** Scheme of classical AMPK-TSC2-mTORC1 signaling pathway and heatmap representing level of corresponding proteins in tumor samples. n = 1 experiment with 9 mice/group. **Q** Western blot (WES) of AMPK and p-AMPK (Thr172). n = 2-5 independent experiments with 3-5 mice/group each. Linear mixed effect model with treatment (HSD vs. NSD) as fixed factor and experimental replicate as random factor. Data are presented as mean ± SEM. **R** Gene expression level and protein level of TSC2 or p-TSC2 (S981). n = 6 independent experiments with 3–8 mice/group each. Linear mixed effect model with treatment (HSD vs. NSD) as fixed factor and experimental replicate as random factor. Data are presented as mean ± SEM. **S** WES of mTORC1 and p-mTORC1 (S2448). n = 3–4 independent experiments with 3–5 mice/group each. Linear mixed effect model with treatment (HSD vs. NSD) as fixed factor and experimental replicate as random factor. Data are presented as mean ± SEM. All analyses were done on day 14 after tumor injection.

To ensure consistency across all performed experiments, we continued to use B16-OVA cells, despite shifting our focus from immune stimulation to the effect on melanoma cells themselves. Next, we investigated the effect of HSD on tumor cell metabolism on a functional level. Puromycin incorporation assays revealed significantly reduced protein synthesis, indicating overall reduced metabolic activity (Fig. 4A). Metabolic flux analysis using the Seahorse XF analyzer showed a remarkable loss of glycolytic and respiratory activity in HSD tumors compared to NSD tumors, as assessed by the extracellular acidification rate (ECAR) and oxygen consumption rate (OCR) (Fig. 4B, C). Suspecting mitochondrial dysfunction as the cause of impaired respiration, we analyzed the gene expression of electron transport chain components (Supplementary Fig. 3A), evaluated mitochondrial membrane potential using JC-1 staining (Supplementary Fig. 3B), assessed mitochondrial activity with the Mitotracker DeepRed probe, a membrane potential-dependent dye (Supplementary Fig. 3C), and measured the enzyme activity of complexes II and III in mitochondria isolated from NSD and HSD tumor cells (Supplementary Fig. 3D). However, none of these analyses revealed any defects in the respiratory chain that would explain impaired cellular respiration. We then hypothesized that the limited cellular metabolism of HSD tumor cells might result from cellular starvation caused by (a) reduced nutrient availability, (b) impaired nutrient uptake, or (c) impaired nutrient processing. To explore these possibilities, we first measured serum glucose and lipid levels, but observed no significant differences overall, despite a slight decrease of triglycerides and increase of free fatty acids in HSD mice (Figs. 4D, 4E). Next, we evaluated the ability of HSD tumor cells to acquire nutrients by assessing mRNA expression of the glucose transporter GLUT1 (Slc2a1), the sodium-dependent glucose transporter NAGT (Slc5a1) and various fatty acid transport proteins (Slc27a1-Slc27a6). We found no differences except for an increase in Slc27a1 expression (Fig. 4F). To confirm the functionality of glucose and lipid transport under HSD, we performed ex vivo nutrient uptake assays using fluorescent glucose and fatty acid analogs (2-NBDG and BODIPY FL-C_16_). We did not observe differences between NSD and HSD tumors, confirming that nutrients were available and equally taken up by NSD and HSD tumors (Fig. 4G, H). However, mRNA expression of glycolysis-related enzymes, was significantly decreased, especially levels of Phosphofructokinase (PfkI), the rate-limiting enzyme of glycolysis, and Phosphoglycerate kinase (Pgk1) (Fig. 4I). This suggests that while HSD tumor cells are able to take up glucose, they might not be able to effectively metabolize it for energy production. To obtain a clearer understanding of the intracellular metabolic processes, we performed metabolic profiling. A total of 652 metabolites were detected, of which 124 were significantly upregulated and 139 significantly downregulated in HSD tumor cells (Fig. 4J). PCA analysis revealed a distinct clustering between NSD and HSD tumors (Fig. 4K) and differences were observed across all metabolic classes, indicating a global shift in cellular metabolism rather than differential regulation of one particular pathway (Fig. 4L). Consistent with our earlier observation of unchanged glucose uptake but reduced expression of rate-limiting enzymes, we detected high intracellular levels of glucose and glucose-6-phosphate, and low levels of fructose-1,6-bisphosphate, a downstream product in glycolysis, in HSD cells (Fig. 4M). This strengthened the hypothesis that carbohydrates are taken up, but their conversion is impaired due to glycolytic enzyme downregulation. Given the decreased rate of mitochondrial respiration and protein synthesis, we also analyzed intracellular amino acid and peptide levels and discovered a strong accumulation of both (Fig. 4N and Supplementary Fig. 4), indicating that amino acids are available, but processing was impaired, similar to the block in glycolysis. Notably, metabolic profiling revealed decreased carnitine levels, which are essential for fatty acid shuttling into mitochondria (Fig. 4O). This potentially explains the loss of mitochondrial respiration, despite fully functional mitochondria. Collectively, these findings suggest that while nutrients are being taken up, they are not adequately processed.

We then investigated whether HSD tumor cells experienced metabolic stress, that would lead to upregulation of 5’ adenosine monophosphate-activated protein kinase (AMPK), a critical regulator of cellular energy levels and metabolic stress [31]. Typically, AMPK upregulation activates TSC2, which in turn leads to inhibition of mTORC1. Mass spectrometry indeed showed a significant increase in TSC2, along with a decrease in downstream proteins like RHEB and MTORC1 (Fig. 4P). Intriguingly, AMPK was not upregulated, suggesting that the cells were not experiencing metabolic stress or nutrient deficiency (Fig. 4P). Western blot validation of total AMPK and phosphorylated AMPK (T172), indicating kinase activity, confirmed similar levels in NSD and HSD tumors (Fig. 4Q and Supplementary Fig. 6). However, Tsc2 mRNA expression and both total TSC2 and phosphorylated TSC2 (S981) protein were increased in HSD tumor cells (Fig. 4R and Supplementary Fig. 6). Protein levels of (phosphorylated) mTORC1 confirmed a significant reduction in mTORC1 activity (Fig. 4S and Supplementary Fig. 6). Overall, these results demonstrate that a HSD suppresses cellular protein synthesis and cell growth through upregulation of TSC2 and inhibition of mTORC1 in a coordinated manner, rather than as a stress response.

**Fig. 5:**
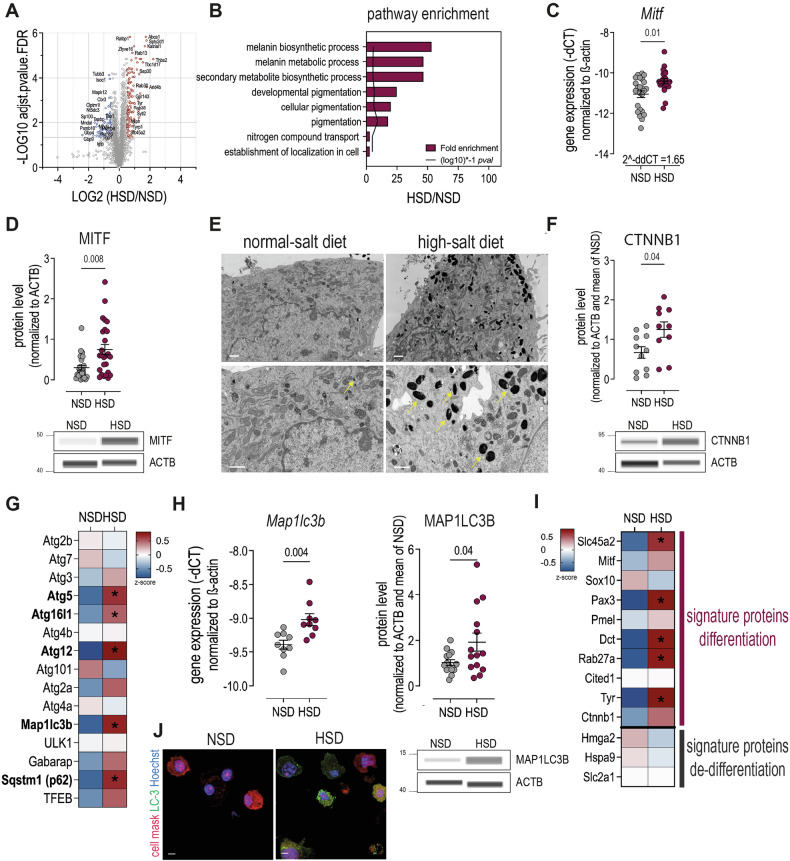
HSD induces melanogenesis and upregulation of differentiation marker. **A** Volcano plot of differentially expressed proteins in tumor cells isolated from mice on HSD vs. NSD (9 mice/group). **B** Pathway enrichment analysis. **C** Gene expression level of *Mitf*. n = 4 independent experiments with 4–9 mice/group each. Linear mixed effect model with treatment (HSD vs. NSD) as fixed factor and experimental replicate as random factor. Data are presented as mean ± SEM. **D** Protein level (WES) of MITF. n = 7 independent experiments with 3–5 mice/group each. Linear mixed effect model with treatment (HSD vs. NSD) as fixed factor and experimental replicate as random factor. Data are presented as mean ± SEM. **E** Representative SEM images of tumor cells isolated from mice on NSD and HSD. Scale bar = 1 µm. **F** Protein level (WES) of beta-catenin. n = 3 independent experiments with 3–6 mice/group each. Linear mixed effect model with treatment (HSD vs. NSD) as fixed factor and experimental replicate as random factor. **G** Z-score of autophagy-related proteins in ex vivo tumor samples (9 mice/group). **H** Gene and protein expression level of total LC-3 in ex vivo tumor samples. n = 1–4 independent experiments with 3–5 mice/group each. Linear mixed effect model with treatment (HSD vs. NSD) as fixed factor and experimental replicate as random factor. Data are presented as mean ± SEM. **I** Z-score of signature proteins for (de-) differentiation of melanoma cells (9 mice/group). **J** B16-OVA tumor cells were isolated on day 14 from tumors grown in mice on NSD or HSD and enriched via magnetic bead labeling (MACS) and stained for LC-3 (green), cell mask (magenta) and DNA/nucleus (Hoechst). All analyses were done on day 14 after tumor injection.

**Fig. 6:**
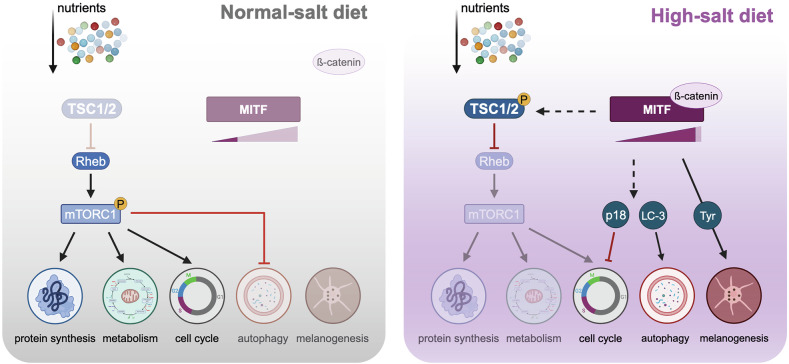
Model of HSD-induced tumor growth suppression. During both, NSD and HSD nutrients are available and taken up by tumor cells. During NSD, MITF- as well as TSC2-levels are low, allowing mTORC1 signaling. This results in enhanced proteins synthesis, metabolic activity and progression through the cell cycle, while autophagy and melanogenesis remain on a basal level. Upon HSD, MITF and TSC2 are upregulated. ß-catenin acts as a co-factor that stabilizes MITF expression, resulting in increased melanogenesis. Due to high TSC2 activity, mTORC1 is downregulated, resulting in shutdown of metabolic activity and protein synthesis as well as cell cycle arrest (p18), accompanied by an increase in autophagy. Red arrows = inhibition; black arrows = induction; black, dashed arrows = proposed/hypothesized activation.

To investigate how HSD induces cell cycle arrest and metabolic standby in melanoma cells, we further analyzed our proteomic data and detected 6197 proteins, with 136 significantly upregulated and 89 downregulated under HSD (Fig. 5A). Enrichment analysis highlighted an upregulation of melanogenesis-related pathways (Fig. 5B). Melanogenesis is regulated by Microphtalmia-Associated Transcription Factor (MITF), thus we measured Mitf mRNA and MITF protein level and found a significant upregulation of both in HSD tumors (Figs. 5C, D and Supplementary Fig. 6). Scanning electron microscopy showed an increased number and larger size of melanosomes in HSD tumor cells, confirming increased melanogenesis (Fig. 5E). MITF is a driver of melanocyte differentiation and oncogenesis and acts as a central hub with various downstream targets [32]. It is triggered by multiple upstream signals and different co-factors that determine its expression level and downstream targets. Primary inducer is alpha-Melanocyte-stimulating hormone (α-MSH), a hormone secreted upon UV exposure, which binds to the Melanocortin-1-receptor (MC1R) and initiates an intracellular signaling cascade that leads to MITF upregulation [33]. We hypothesized that HSD would increase Proopiomelanocortin (POMC), the precursor of α-MSH, and Adrenocorticotropic hormone (ACTH), which is known to be regulated by salt [34]. Indeed, we found increased levels of POMC and mature ACTH in the hypothalamus and pituitary gland of tumor-bearing HSD mice (Supplementary Fig. 5A, B), however, we neither detected increased serum levels of α-MSH or accumulation of α-MSH in the tumor microenvironment, nor was MC1R upregulated in HSD tumor cells (Supplementary Fig. 5C–F), excluding this pathway as trigger for upregulation of MITF. Next, we measured levels of ß-catenin (CTNNB1), known to stabilize MITF and enhance melanogenesis [35]. We detected increased β-catenin levels in HSD tumors, suggesting that it also plays a role in activation and stabilization of MITF under HSD (Fig. 5F and Supplementary Fig. 6). MITF also regulates autophagy and lysosome biogenesis [36]. Since autophagy is induced by mTORC1 inhibition [37], associated with melanocyte differentiation [38] and previously shown to be triggered by HSD in muscle and liver cells [39], we hypothesized that it is also triggered in HSD melanoma, to maintain basal cellular maintenance. Our proteomic data revealed a significant increase of various autophagy-related proteins upon HSD (Fig. 5G), which we confirmed by increased expression of LC-3 (Map1lc3b) mRNA and protein in ex vivo tumor samples (Fig. 5H, J and Supplementary Fig. 6). As autophagy regulates stemness and differentiation [40] and MITF drives melanocyte differentiation, we hypothesized that HSD could trigger melanoma re-differentiation and quiescence. To test this, we analyzed the expression of proteins recognized as signature markers for melanoma differentiation and de-differentiation [4, 9, 41, 42]. We found a significant increase of Slc45a2, Pax3, Dct, Rab27a and Tyr under HSD, indicating differentiation, while the expression of Hmga2, Hspa9 or Slc2a1, indicative of de-differentiation remained unchanged (Fig. 5I). Overall, this suggests that a HSD might reverse the transition from a melanocyte to a melanoma cell, entering cell cycle arrest and reducing nutrient requirements as they adopt a standby mode, similar to non-malignant melanocytes (Fig. 6).

## Discussion

The present study shows that a HSD inhibits tumor growth in a mouse model of melanoma by shifting cells back into a re-differentiated state, characterized by cell cycle arrest, metabolic shutdown, and increased melanogenesis. The growth inhibitory effect of HSD on melanoma and other cancer cell types has been observed previously and was explained by stimulation of the anti-tumor immune response, due to accumulation of sodium in the tumor and the surrounding tissue [21, 22]. However, in our setting, we failed to detect sodium accumulation in HSD mice compared to normal salt conditions, and consistently no enhanced anti-tumor immune response. Also, treatment of B16-OVA cells with 40 mM NaCl in-vitro did not recapitulate the phenotype observed in vivo (Supplementary Fig. 9A–D). Furthermore, we did not detect an increase in serum hippurate level or translocation of Bifidobacteria that have been observed in other studies [25] (Supplementary Fig. 8B, C). While this does not argue against sodium-mediated immune stimulation in other settings, it clearly indicates that a HSD has additional, molecularly unidentified anti-tumor effects on melanoma cells. Importantly, growth of Y-1 cells (adrenal-derived cancer cells) remained unaffected by the HSD, further strengthening that the impact of the diet appears to be specific to melanoma cells and therefore likely driven by regulation of a melanoma-specific factor. These findings were further corroborated by using another melanoma cell line (B16V) that does not express OVA (Supplementary Fig. 10). We observed a strong upregulation of MITF, a key regulator of melanoma metabolism, differentiation, and melanogenesis [43, 44], in tumor cells from HSD mice. Classically, melanogenesis is activated upon a-MSH stimulation. However, in our model, this signaling pathway did not trigger MITF activation. It would be interesting to determine whether HSD also affects other non-melanoma cancer cells, such as renal cell carcinoma (RCC), which harbors aberrant non-melanoma specific isoforms of MITF [45–47]. One target of MITF and key enzyme for melanin production is tyrosinase. Recently it was shown that tyrosine injections into tumors of melanoma mice activated tyrosinase, reduced phosphoglucomutase (PGM), a glycolysis enzyme, and consequently slowed down tumor growth [48]. In contrast, we observed a broader glycolytic shutdown on the transcriptional level, suggesting that HSD-induced melanogenesis impacts more than a single glycolytic enzyme. MITF is essential for melanocyte development [7] and its role in melanoma progression has been debated due to its seemingly opposing effects on proliferation. The rheostat model resolved these discrepancies and explained metabolic and phenotypic variability in melanoma [5, 8]. Regulated by diverse signals and co-factors, MITF controls over 100 target genes [32]. We observed increased levels of ß-catenin, a co-factor shown to initiate a feedback loop with MITF, enhancing melanogenesis, while limiting Wnt-signaling [35]. This supports our conclusion that HSD enhances melanogenesis and slows tumor growth. Excessively high levels of MITF were shown to induce p21 and p16, both members of the INK4 family of CDK inhibitors, causing cell cycle arrest and reduced metabolic activity [10]. We found that HSD upregulated p18, another inhibitor of CDK4/6 [49], which could be driven by MITF. Cancer cells often bypass metabolic restrictions by increasing nutrient uptake and shifting to enhanced glycolysis (Warburg effect), and melanoma cells also increase their mitochondrial activity to generate sufficient energy to fuel their growth and proliferation [50]. We observed that both, glycolysis and oxidative phosphorylation, were reduced in HSD tumor cells, driven by upregulation of TSC2 and subsequent mTORC1 inhibition. Importantly, TSC2 is often induced by starvation [51], while upregulation in our model was independent of starvation, dysfunctional mitochondria, or any stress response. This suggests a coordinated reduction of metabolic activity to basal levels, a characteristic of quiescent, differentiated cells [52], possibly also driven by MITF. Autophagy, essential for cellular homeostasis, supports survival, development, and differentiation, including the transition from melanoblasts to melanocytes [40, 53, 54], and MITF overexpression was shown to modestly increase autophagosome numbers even in the absence of starvation in-vitro [55]. Consistent with earlier studies in muscle and liver [39], we observed elevated expression of autophagy-related proteins in HSD melanoma cells, providing additional evidence for a regulated shift from uncontrolled metabolic activity of malignant cells to basal metabolic homeostasis. It was previously demonstrated that melanoma development entails a de-differentiation process from melanocytes towards a stem-cell/neural crest-like state [9]. Four distinct stages have been identified, each marked by the expression of specific signature genes. In our model, we observed an increased expression of differentiation markers in HSD tumor cells. Together, the elevated expression of MITF, associated with growth arrest and metabolic shutdown, as well as the expression of differentiation marker suggest that a HSD leads to re-differentiation of melanoma cells. A limitation of our study is that the molecular mechanisms by which a HSD induced MITF expression and its downstream targets in melanoma have not been fully deciphered. While we analyzed the effects on the cell cycle and metabolic outcomes, the systemic trigger(s) for MITF expression remain to be identified in future studies. The normal sodium concentrations in and around the tumor indicates that the regulatory mechanisms under HSD are more complex than mere tissue sodium storage. Future studies need to directly measure sodium content, rather than assuming that it is increased. In conclusion, we demonstrate that a HSD reduces melanoma growth independent of the immune response, by upregulating MITF, p18, TSC2, and differentiation markers. These findings suggest that HSD may not generally support all anti-tumor therapies by invigorating immunity.

## Material and methods

### Mouse experiments

All mice (C57BL/6J, Rag2tm1.1Cgn, B6.129(Cg)-Ccr2tm2.1Ifc/J) were bred and housed under specific pathogen-free (SPF) conditions at the animal facility of the University Hospital Bonn (House of Experimental Therapy, HET). Female mice aged 8–12 weeks were used for all in vivo experiments. All animal procedures complied with the Federation of European Laboratory Animal Science Associations (FELASA) guidelines and were approved by the relevant German authorities. Mice were randomly assigned to groups fed either a normal salt diet (NSD) (0.5% sodium chloride, ssniff Spezialdiäten Cat. E15430-047) and received autoclaved tap water (13.3 ± 1.6 mg/l NaCl = 0.1%) (water analysis 2024, municipal utilities, available at: https://www.stadtwerke-bonn.de/fuer-zuhause/produkte/wasser/wasseranalyse/), or a high salt diet (HSD) (4% sodium chloride, ssniff Spezialdiäten Cat. E15431-34) with 0.9% sodium chloride in autoclaved tap water, provided ad libitum for 3 weeks, starting 7 days before tumor injection. For the skin tumor model, the injection site was shaved, and 0.5 * 10^6^ B16-OVA melanoma cells or Y-1 cells were administered subcutaneously (s.c.) in PBS. Tumor growth was tracked by measuring the length (L) and width (W) of the tumors. Tumor volume was calculated using the formula: V(mm^3^) = L(mm) × W(mm)^2^ × 0.5. In the lung metastasis model, 0.5 * 10^6^ B16-OVA-Luciferase-expressing melanoma cells were injected intravenously (i.v.) in PBS. Tumor progression in the lung was assessed by in vivo luminescence imaging using the IVIS Lumina LT Series III (Caliper Life Science). Mice were anesthetized with isoflurane, and 4.5 mg of luciferin (BioVision, 792-10 G) was injected intraperitoneally (i.p.), followed by in vivo imaging of luciferase activity after 5 min of incubation. Lymphocyte depletion was achieved by administering 400 µg/mouse of anti-Thy1 (BioXCell, #BE0076) i.p. on day 0, followed by 200 µg/mouse every 3 days. For NK cell depletion, mice received 1.2 mg/kg anti-NK1.1 (BioXCell, #BE0036) i.p. weekly, starting 7 days before tumor injection. Neutrophil depletion was induced with 500 µg/mouse of anti-Ly6G (BioXCell, #BE0075-1) i.p. every 3 days, beginning on day 0. To deplete macrophages, 200 µl of clodronate liposomes (Liposoma, #C-005) was administered i.p. every 3 days starting on day 0. All depletion antibodies were diluted in PBS. The number of mice (sample size) was determined based on an a priori power analysis conducted using G*Power analysis as well as previous experience with the same model in our institute. In line with ethical standards, the number of animals used was minimized while still ensuring the reliability of the results and maintaining statistical validity.

### Sample collection tissues

Mice were euthanized by CO_2_ inhalation on day 14 after tumor injection. Skin tumors, excluding connective tissue, were harvested and either immediately frozen or digested and homogenized at 37 °C in RPMI medium containing 10% DNase/Collagenase. For lung analysis, lungs were fully removed, and metastases were either manually counted or the lung tissue was digested and homogenized in the same medium as the skin tumors for gene expression analysis of Ovalbumin. Single-cell suspensions were passed through a 100 µm nylon filter, and cells were counted in a Neubauer chamber after Trypan blue staining. Cells were then diluted in cell culture medium for further assays. When necessary, tumor cells were isolated from the homogenates using the Tumor Cell Isolation Kit from Miltenyi Biotech (#130-110-187). Hypothalamus and pituitary gland tissues were removed and immediately frozen in liquid nitrogen for subsequent analysis.

### Sample collection blood

Blood was collected from mice from the heart on day 14 after euthanasia. Samples were centrifuged, and serum stored for cytokine analysis.

### Generation of luciferase and KO cell lines

Wild-type B16-OVA cells were used for in vivo experiments. B16-OVA cells expressing firefly luciferase were constructed using Fugene® transfection reagent (Promega) to transfect 293 T cells with retroviruses expressing pRP-mNeon-ffluc2. The supernatant containing ecotrophic retroviruses was collected after 72 h and filtered through a 0.45 μM filter before being incubated with B16-OVA cells. Positive clones were selected on puromycin. NFAT5^−/−^ and SGK1^−/−^ cells were generated as follows: For CRISPR/Cas9 based gene targeting annealed DNA oligos (sgRNA target sites: Nfat5, cctagagaaacatctgtagc; Sgk1, gcttttatgaaacagaga) were cloned into PX458 (Addgene #48138). Plasmids were sequenced and transfected with FuGENE HD (Promega) according to manufacturers’ protocols. Successfully transfected GFP-positive cells were enriched by cell sorting and re-seeded for the generation of monoclones. Established Nfat5^−/−^ and Sgk1^−/−^ B16-OVA monoclones were validated by MiSeq-sequencing.

### Cell culture

All B16-OVA cells (Merck Sigma-Aldrich #SCC420) as well as B16V cells (DSZM #ACC370) were cultured in RPMI 1640 GlutaMAX^TM^ medium containing 10% fetal calf serum (FCS) and 0.1 mg/ml Geneticin (G418, US Biological Life Science) to maintain OVA expression at 37 °C, 5% CO2. The Y-1 cell line was obtained from the American Type Culture Collection (#CCL-79) and also cultured in RPMI 1640 GlutaMAX^TM^ medium containing 10% fetal calf serum (FCS).

### Fatty acid uptake assay

Ex vivo tumor cells were starved for 1 h in PBS + 20 µM Bovine Serum Albumin (BSA) at 37 °C, 5% CO_2_. 5 µM of fluorescent BODIPY™ FL C_16_ (ThermoFisher Scientific #D3821) was added to the cells and incubated for 45 min at 37 °C, 5% CO_2_. Cells were washed and stained for flow cytometric analysis.

### Glucose uptake assay

Ex vivo tumor cells were starved for 1 h in PBS + 10% FCS at 37 °C, 5% CO_2_. After starvation, 100 mM of 2-(N-(7-Nitrobenz-2-Oxa-1,3-Diazol-4-yl)Amino)-2-Desoxyglucose (2-NBDG) (AAT Bioquest, #186689-07-6) was added, and cells were incubated for 45 min at 37 °C, 5% CO_2_. Afterwards, cells were washed and stained for flow cytometric analysis.

### Seahorse assay

Ex vivo tumor cells were seeded in Seahorse XF96 Cell Culture Microplates (Agilent). After attachment of the cells, bicarbonate-free media were added, and the oxygen consumption rate (OCR) and extracellular acidification rate (ECAR) were measured using the Seahorse XFe Analyzer (Agilent) and following the manufacturer´s instructions.

### Flow cytometry

Single cell suspensions obtained from skin tumors were centrifuged and stained in PBS supplemented with 0.1% BSA and 0.1% sodium azide (Carl Roth) for 45 min at 4 °C using fluorochrome-labeled monoclonal antibodies from BioLegend, BD Biosciences, or Invitrogen. Dead cells were excluded from the analysis using the fixable viability dye eFluor780 from ThermoFisher Scientific. For intracellular staining, cells were fixed and permeabilized using the FOXP3/Transcription Factor Staining Buffer Set (eBioscience) following the manufacturer’s instructions. Afterwards, samples were measured using BD FACS Canto II (BD Biosciences) or BD LSR Fortessa (BD Biosciences) and analyzed with FlowJo software V10.10.0. Antibodies and dyes used are summarized in Table 1.

**Table 1:** Antibodies and dyes.

Antibody	Fluorophore	Company	Cat. nr.
B220	PE	eBioscience	12-0452-83
CD103	PerCP-Cy5.5	BioLegend	121416
CD11b	APC	eBioscience	17-0112-83
CD11b	AF700	BioLegend	101222
CD11c	BV421	BD Biosciences	562782
CD11c	APC	BD Biosciences	550261
CD11c	BV737	BD Biosciences	612796
CD11c	BV711	BioLegend	117349
CD127	APC	BioLegend	135011
CD25	PE	eBioscience	12-0251-83
CD25	APC	Biolegend	102012
CD25	BV510	BioLegend	102011
CD3e	PE-Cy7	Biolegend	100219
CD3e	Fitc	BioLegend	100306
CD4	PE-Cy7	Biolegend	100422
CD4	BV421	Biolegend	100438
CD45	BV421	Biolegend	103134
CD45	BUV395	BD Biosciences	564279
CD8a	BV510	Biolegend	100752
CD8a	BV605	BioLegend	100744
F4/80	PE	Biolegend	123110
FoxP3	FITC	eBioscience	11-5773-82
gd-TCR	PerCP-Cy5.5	Biolegend	118120
IL-17A	APC	BioLegend	506916
Ly6C	PerCP-Cy5.5	BioLegend	128012
Ly6C	BV605	BioLegend	128035
Ly6G	APC	BioLegend	127614
Ly6G	FITC	BioLegend	127606
Ly6G	BV421	Biolegend	127628
MHCII	FITC	Biolegend	107606
NK1.1	APC	BioLegend	108710
NK1.1	AF700	Biolegend	156512
TCRb	BV421	BioLegend	109230
TCRb	APC	Biolegend	109212
Fixable Viability Dye		eBioscience	65-0865-14

### Isolation of mitochondria and respiratory chain activity assays

For mitochondrial isolation from tumor cells, tumor tissues were harvested, and tumor cells were isolated and enriched with the tumor cell isolation kit as described above. All steps were performed at 4 °C/on ice within ca. 2 h. These isolated tumor cells were processed as described previously [56]. Briefly, tumor cells were mixed with solution A (20 mM HEPES/KOH (pH 7.6) + 220 mM D-Mannitol + 70 mM sucrose + 1 mM EDTA + 0.5 mM PMSF in PBS) and homogenized. Gradual centrifugation resulted in isolated mitochondria that were resuspended in solution B (solution A + 2 mg/ml BSA), and the total concentration was measured. The isolated mitochondria were used to measure the activity of complex II, III, or combined complex II + III as described [57] with the following modifications: measurements were performed in 96-well plates using a microplate reader (Infinite M Plex, Tecan, Männedorf, Switzerland) and potassium succinate was used as substrate for complex II and II + III activity assays.

### Lipidomic analysis

All solvents were HPLC grade or LC-MS grade, from VWR International GmbH (Darmstadt, Germany), Merck KGaA, Darmstadt, Germany. Lipid extraction from serum samples was done as follows: To a 2 μL murine serum sample, 500 μL of extraction mix was added. The extraction mix was prepared in CHCl₃/MeOH (1:5) and contained the following internal standards: 250 pmol PE (31:1) d7, 472 pmol PC (31:1) d7, 98 pmol PS (31:1), 84 pmol PI (34:0), 56 pmol PA (31:1), 51 pmol PG (28:0), 28 pmol CL (56:0), 39 pmol LPA (17:0), 35 pmol LPC (17:1), 38 pmol LPE (17:0), 32 pmol Cer (17:0), 240 pmol SM (17:0), 55 pmol GlcCer (12:0), 339 pmol TG (50:1-d4), 111 pmol CE (17:1), 64 pmol DG (31:1), 103 pmol MG (17:1), 724 pmol Chol (d6), and 45 pmol Car (15:0). Samples were sonicated in the bath sonicator for 10 s and subsequently lipids were collected. After centrifugation at 20,000 × *g* for 2 min, supernatants were retrieved and mixed with 200 μL CHCl3 and 800 μL 1% acetic acid. The sample were briefly shaken and centrifuged for 2 min at 20,000 × *g*. The upper aqueous phase was removed, and the entire lower phase was transferred into a new tube and evaporated in a speed vac (45 °C, 10 min). Spray buffer (8/5/1 2-propanol/MeOH/water, 10 mM ammonium acetate) was added and samples were sonicated for 5 min. For lipidomics analysis, all samples were separately infused at 10 μl/min into a Thermo Q Exactive Plus spectrometer equipped with the HESI II ion source for shotgun lipidomics. MS1 spectra (resolution 280,000) were recorded in 100 m/z windows from 250 to 1200 m/z (positive mode), followed by recording MS/MS spectra (resolution 70 000) by data-independent acquisition in 1 m/z windows from 250 to 1200 (positive mode). Raw files were converted to .mzml files and imported into and analyzed by LipidXplorer software using custom mfql files to identify sample lipids and internal standards. For further data processing, absolute amounts were calculated using the internal standard intensities.

### RNA isolation and qPCR

RNA of tumor samples was isolated with Nucleo Spin RNA kit (Macherey-Nagel). cDNA was generated using a High-Capacity cDNA Reverse Transcription Kit (Life Technologies, #4373966). Oligonucleotides were purchased from Qiagen or Invitrogen. SYBR Green was used from Applied Biosystems (#C14512). Quantitative real-time PCR was performed with Quant Studio 6, Applied Biosystems. Oligonucleotides used are summarized in Table 2.

**Table 2:** Oligonucleotides.

Gene	Forward	Reverse
OVA	5-GTGTTTAGCTCTTCAGCCAATCT-3	5-CTGCATGGACAGCTTGAGATA-3
HK1	5-CTTCGACAAAGTGGTGGACG-3	5-TTACGGACGATCTCACCCAG-3
HK2	5-CCCTGTGAAGATGTTGCCCAC-3	5-TGCCCATGTACTCAAGGAAGT-3
PFKM	5-GGGTCATGTACAGCGAGGA-3	5-GGCCTCCATACCCATCTTG-3
ALDOA	5-AGGAGGAGTACATCAAGCGC-3	5-TGGGAAAGAGCCTGAAGACC-3
PGK1	5-AAGACTGGCCAAGCTACTGT-3	5-AATCTGCTTAGCTCGACCCA-3
PGM1	5-CAACAGTGATCCGGAGCATC-3	5-TGTTCTTCAATGGCACCGAC-3
ENO1	5-ACCACAACCTGAAGAACGTG-3	5-GAATCCACCCTCATCACCCA-3
SLC2A1	5-GCTGTGCTTATGGGCTTCTC-3	5-CACATACATGGGCACAAAGC-3
SLC5A1	5-TCTGTAGTGGCAAGGGGAAG-3	5-ACAGGGCTTCTGTGTCTTGG-3
CD36	5-TTCTCATGCCAGTCGGAGAC-3	5-AAGACACAGTGTGGTCCTCG-3
SLC27A1	5-ATTGTGGTGCACAGCAGGTACTA-3	5-TGGTAGAGTGGCAGGCAGTCA-3
SLC27A2	5-GCGTGCCTCAACTACAACATT-3	5-CCTCCTCCACAGCTTCTTGT-3
SLC27A3	5-GAGAACTTGCCACCGTATGC-3	5-GGTCTCAGTAGTGCCAAAGA-3
SLC27A4	5-CTATGACTGCCTCCCCCTCT-3	5-CATGCCGTGGAGTAAGCAC-3
SLC27A5	5-GCCTTCGAGCTACGATTAAGTG-3	5-GATAAGATGGCTGGCTTTGG-3
SLC27A6	5-CAACACCTGTGAACCCACTG-3	5-TCTTCTATGCTTCCAAGCAAATCT-3
MITF	5-ATGCTGGAAATGCTAGAATATAATC-3	5-CAATCAGGTTGTGATTGTCC-3
MAP1LC3	5-GACCAGCACCCCAGTAAGAT-3	5-TGGGACCAGAAACTTGGTCT-3
LDHA	5-ATGAAGGACTTGGCGGATGA-3	5-CGCCCTTGAGTTTGTCTTCC-3
PDHA1	5-TACGGACCACCTCATCACTG-3	5-CAACCTCCTCTTCGTCCTGT-3
PDHB	5-CAGCTAGTAGAGGACACGGG-3	5-TGAAAACGCCTCTTCAGCAG-3
TPI	Qiagen	#QT00104167
GAPDH	Qiagen	#QT00199388
ACTB	Qiagen	#QT00095242

### Transcriptomics

Tumors were harvested on day 14 as usual. The 3’ mRNA-seq library was prepared with the forward QuantSeq 3’ mRNA-Seq Library Prep Kit for Illumina (Lexogen GmbH, Austria) according to the manufacturer’s protocol. The minimum amount of RNA required was 50 ng/sample with a RIN ≥ 7. Size distribution and yield of the 3’ mRNA-seq were measured with the D1000 high-sensitivity tape station (Agilent) prior to pooling of the barcoded libraries. The pooled 3’ mRNA-Seq libraries were loaded on the Illumina HiSeq2500 platform and analyzed by a 50-cycle high-output run with a sequencing depth of 10 M by the UKB NGS core facility, University of Bonn. The average read length after alignment was 51 bp.

Raw data are deposited to the NCBI GEO archive (GSE280927). FASTQ files were aligned to the mm10 mouse reference genome with StarAligner. Further computational 3’ mRNA-seq analysis was performed with the Bioconductor/R computing environment. The voom method of the limma package was used for normalization and linear modeling with quantile normalization. mRNA expression values were obtained by log2 transformation of the read counts per million (cpm) with the voom algorithm. Differentially expressed genes were identified by an empirical Bayes moderated *t*-test statistic using limma package. The hallmark gene set collection, as well as reactome gene set collection provided by the Molecular Signature Database (MSigDb) from the Broad Institute was used for gene set enrichment and reactome analysis [58]. Reactome network visualization was done with Cystoscope software and Adobe Illustrator.

### Metabolomics

Skin tumors of mice were harvested on day 14 after tumor injection and immediately stored in liquid nitrogen. Analysis of metabolites within the tumor samples was carried out by METABOLON, INC. (Morrisville, NC).

### Ion concentration in tissues

Tumor, skin, and lung tissues from tumor-bearing mice fed NSD or HSD for 14 days were harvested and immediately frozen in liquid nitrogen. Atomic absorption spectrometry (AAS) was used to determine the sodium content. Tissues were dried at 70 °C for 72 h to determine water content, incinerated at 550 °C, and resuspended in 2 ml 5% nitric acid (X898.2, Carl Roth). Samples were diluted with 0.5% caesium chloride (1.02039, Sigma Aldrich) and mixed by inverting. The diluted samples were analysed using the Thermo Scientific iCE 3000 Series AA Spectrometer (Thermo Scientific).

### Western blot

Single cell suspensions or tissue homogenate were lysed in RIPA lysis buffer (Sigma-Aldrich #R0278) containing Protease inhibitor (ROCHE cOmplete™ Proteasehemmer #11697498001) and Phosphatase inhibitors (ROCHE PhosSTOP™ #4906845001). Then, protein concentration was determined with the BCA assay. Protein levels were determined using Simple Western™ technologies (Biotechne), combining size-based capillary electrophoresis with immunodetection via primary antibodies. For this, 1 µg protein was loaded into capillaries according to manufacturer´s instructions. Data were analyzed with the Software Compass for Simple Western™. Following antibodies were used: AMPK a1 (BioTechne, #MAB3197), p-AMPK (Thr172) (Cell Signaling, #50081T), Tuberin/TSC2 (Cell Signaling, #4308T), and p-TSC2 (S981) (Invitrogen, #PA5-118723), mTOR (Cell Signaling, #2971S), p-mtor (S2448) (Invitrogen, #44-1125G), b-catenin (Novus, #NBP1-54467), MITF (Cell Signaling, #12590S), POMC (Cell Signaling, #23499S), LC3B I/II (Sigma-Aldrich, #L7543), beta actin (Cell Signaling, #4970S), NFAT5 (Novus, #NB120-3446), SGK1 (Cell Signaling, #12103) and Mc1r (Invitrogen, #PA5-21911).

### Proteomics

Ex vivo tumor cells were lysed and denatured with 2% SDS, 50 mM Tris-HCl pH 8, 150 mM NaCl, 10 mM TCEP, 40 mM chloroacetamide, and protease inhibitor cocktail tablet (EDTA-free, Roche) at 95 °C for 5 min, then sonicated for 1 min (1 s ON/ 1 s OFF pulse, 45% amplitude) and boiled again for 5 min at 95 °C. Pure proteins were obtained with methanol/chloroform precipitation. Protein pellets were then resuspended in 8 M urea solution containing 10 mM EPPS. 100 μg of protein per samples were digested overnight at 37 °C with LysC (Wako Chemicals) at 1:50 (w/w) ratio and Trypsin (Promega, V5113) at 1:100 (w/w) ratio. Digested peptides are purified using Sep-Pak tC18 cartridges (Waters, WAT054955). Peptide concentrations were determined with a μBCA assay (ThermoFisher Scientific, 23235), and 10 μg of peptide per sample was labeled with TMTpro 18-plex reagents (ThermoFisher Scientific, A52045) in a 1:2.5 (w/w) ratio (2.5 µg TMT reagent per 1 µg peptide). TMT labeled samples were adjusted to equal amounts and pooled. Then the pool was fractionated into eight fractions using the High pH Reversed-Phase Peptide Fractionation Kit (Thermo Fisher Scientific, 84868) according to the manufacturer’s protocol and dried.

Fractions were then resuspended in 2% acetonitrile and 0.1% formic acid and separated on an Easy nLC 1200 (ThermoFisher Scientific) and a 22 cm long, 75 μm ID fused-silica column, which had been packed in-house with 1.9 μm C18 particles (ReproSil-Pur, Dr. Maisch) and kept at 50 °C using an integrated column oven (Sonation). Assuming equal amounts in each fraction, 1 µg of peptides were eluted by a non-linear gradient adapted for each fraction (F1: 3–24, F2: 4–26, F3: 5–28, F4: 6–30, F5: 7–32, F6: 8–34, F7: 9–36, F8: 10–38% B) over 150 min followed by a step-wise increase to 75% ACN in 6 min which was held for another 9 min. After that, peptides were directly sprayed into an Orbitrap Fusion Lumos mass spectrometer equipped with a nanoFlex ion source (ThermoFisher Scientific). Sprayed peptides were analysed Multi-Notch MS3-based TMT method in order to minimize ratio compression and ion interference as previously described [59] for total proteomics. Full scan MS spectra (350–1400 m/z) were acquired with a resolution of 120,000 at m/z 200, maximum injection time of 100 ms, and AGC target value of 4 × 10^5^. The most intense precursors with a charge state between 2 and 5 per full scan were selected for fragmentation (“Top Speed” with a cycle time of 1.5 s) and isolated with a quadrupole isolation window of 0.7 Th. MS2 scans were performed in the Ion trap (Turbo) using a maximum injection time of 50 ms, AGC target value of 1.5 × 10^4^ and fragmented using CID with a normalized collision energy (NCE) of 35%. SPS-MS3 scans for quantification were performed on the 10 most intense MS2 fragment ions with an isolation window of 0.7 Th (MS) and 2 m/z (MS2). Ions were fragmented using HCD with an NCE of 50% and analysed in the Orbitrap with a resolution of 50,000 at m/z 200, scan range of 110–500 m/z, AGC target value of 1.5 × 10^5^ and a maximum injection time of 86 ms. Repeated sequencing of already acquired precursors was limited by setting a dynamic exclusion of 45 s and 7 ppm, and advanced peak determination was deactivated. Raw data were analyzed with Proteome Discoverer (PD) version 2.4 (ThermoFisher Scientific). MS2-spectra were searched against the mouse reference proteome (Taxonomy ID 10090, UniProt, 12-March-2020; “One Sequence Per Gene”, 21959 sequences) and common contaminants (244 entries from MaxQuant’s “contaminants.fasta”) using SequestHT. Precursor mass tolerance was set to 7 ppm and fragment mass tolerance to 0.5 Da after recalibration using the Spectra RC-node with default settings. Static modifications included TMTpro labeling at N-termini and lysines (+304.207) and carbamidomethylation on cysteines (+57.021). Dynamic modifications comprised methionine oxidation (+15.995) and N-terminal acetylation (+42.011). Peptide spectrum matches (PSMs) were filtered using Percolator with FDR < 1%. For quantification, only PSMs with signal-to-noise >10 and co-isolation <50% from unique peptides were considered after total intensity normalization. Only high confident proteins (combined *q*-value < 0.01) were used for downstream analyses. Protein file from PD was then exported to Excel for further processing. Normalized abundances from protein file were used for statistical analysis after contaminations and complete empty values were removed. Statistical significance was determined using two-sided, unpaired Student’s *t*-tests (*p*-value < 0.05) with log2 fold-change cut-off (≥0.5 or ≤−0.5) in R version 4.0.2 in RStudio. The mass spectrometry proteomics data have been deposited to the ProteomeXchange Consortium via the PRIDE [60] partner repository with the dataset identifier PXD057160.

### Electron microscopy

Ex vivo tumor samples were washed and resuspended in serum-free medium containing 2.5% Glutaraldehyde for fixation. After 2 h, samples were washed in PB-glutaraldehyde buffer and further processed for SEM by the Microscopy Core facility of the University of Bonn.

### Cytokine measurement

Serum cytokines were measured with the Legendplex Kit from Biolegend (#740150) according to the manufacturer’s instructions after sacrificing mice on day 14 after tumor injection.

### Histology

Fibrotic condition of tumor tissue was evaluated using Sirius Red staining on paraffin-embedded tissue sections. Tumors and the surrounding skin were collected and incubated in 4% methanol-free PFA in PBS at 4 °C overnight. The following day, the samples were rinsed for 4 h, gradually dehydrated, and embedded in liquid paraffin. Tumor and skin sections were cut into 2 μm thick slices using a rotary microtome and dried overnight at 60 °C before staining. After deparaffinization and rehydration, the slides were stained with Pico Sirius Red solution (in saturated picric acid) for 1 h, washed with acidified water, and dried. After gradual dehydration, slices were fixed in ROTI® Histokitt. Scanning was performed at 20x and 40x magnifications using a bright field scanner, and the fibrotic areas were visualized and quantified using the Fiji ImageJ software.

### a-MSH ELISA serum/tumor tissue

$\alpha$-MSH levels were quantified in the serum and homogenized tumor homogenate by ELISA according to the manufacturer´s description (Abexxa, #Abx254513).

### Glucose ELISA serum

Serum glucose levels were determined with the Mouse Glucose Assay Kit according to the manufacturer´s description (Crystal Chem, #CC-81692)

### Immunofluorescence staining

Tumors were harvested on day 14 after injection, and tumor cells were isolated via MACS as described above. Cells were then fixed in 4%PFA for 15 min at RT, before permeabilization with 0.1% Triton-X 100 for 10 min. After blocking with 1% BSA-PBS for 30 min at RT, cells were incubated for 3 h with the primary antibody (LC3B I/II (Sigma-Aldrich, #L7543)) and washed 3x 5 min with PBS. Next, cells were incubated for 1 h with the secondary antibody (anti-rabbit-AF488 (Invitrogen #A27034); cell mask (Thermo Fisher Scientific #H32721) and Hoechst (Thermo Fisher Scientific #1399)), washed 3 × 5 min in PBS and mounted in Vectashield (Biozol # H-100). Images were acquired on the ZEISS LSM510 and processed with ImageJ.

### Statistics

The choice of statistical methods was based on the design and structure of each analysis. Two-tailed unpaired *t*-tests were used when comparing two independent groups to assess whether there was a statistically significant difference between their means. One-way ANOVA was applied for comparisons involving more than two groups or multiple levels of single factors, while interaction between two independent variables and their effects on a dependent variable a two-way ANOVA was used. Additionally, we used linear mixed-effects models (LMMs) to account for both fixed effects (e.g., treatment) and random effects (e.g., inter-experimental variability). All analyses were preceded by assessments to confirm normal distribution.

### Software

GraphPad Prism V10 was used to perform all statistical testing, unless stated otherwise. The graphical abstract was created with BioRender.com. Flow cytometry data were analyzed with FlowJo V10.10.0. Cytoscape V3.10. was used for visualization of Reactome networks. Adobe Illustrator V29.0. was used to create all figures.

## Supplementary information

HSD_melanom_CDD_X4_supplementary file

Original Data_western blot

## Data Availability

The proteomics data generated in this study have been deposited to the ProteomeXchange Consortium via the PRIDE partner repository (identifier PXD057160). The 3´mRNAsequencing data have been deposited to the NCBI GEO database (identifier GSE280927). Mass spectrometric raw data for metabolomics/lipidomics are available upon request from the corresponding authors. All other data are available in the main text or the supplementary materials.
