# Supplementary material for: High-salt diet induces immune-independent re-differentiation, metabolic shut down and cell cycle arrest of melanoma
Source: Cell Death Dis. 2025 Dec 20;17(1):102. doi: 10.1038/s41419-025-08329-x (PMC12847697; doi:10.1038/s41419-025-08329-x)
Supplement: Supplementary file 1 — HSD_melanom_CDD_X4_supplementary file [file 41419_2025_8329_MOESM1_ESM.docx]

Supplementary Materials for

**High-salt diet induces immune-independent re-differentiation,**

**metabolic shut down and cell-cycle arrest of melanoma**

Clivia Lisowski*, Natascha E. Stumpf*, Katarzyna Jobin* *et al.*

*Authors contributed equally

†Corresponding author(s) Email: ckurts(at)uni-bonn.de; cliso(at)uni-bonn.de

**This file includes:**

- Figs. S1 to S10

Fig. S1. Reduced tumor growth is not mediated by sodium accumulation or osmotic stress sensing.

**
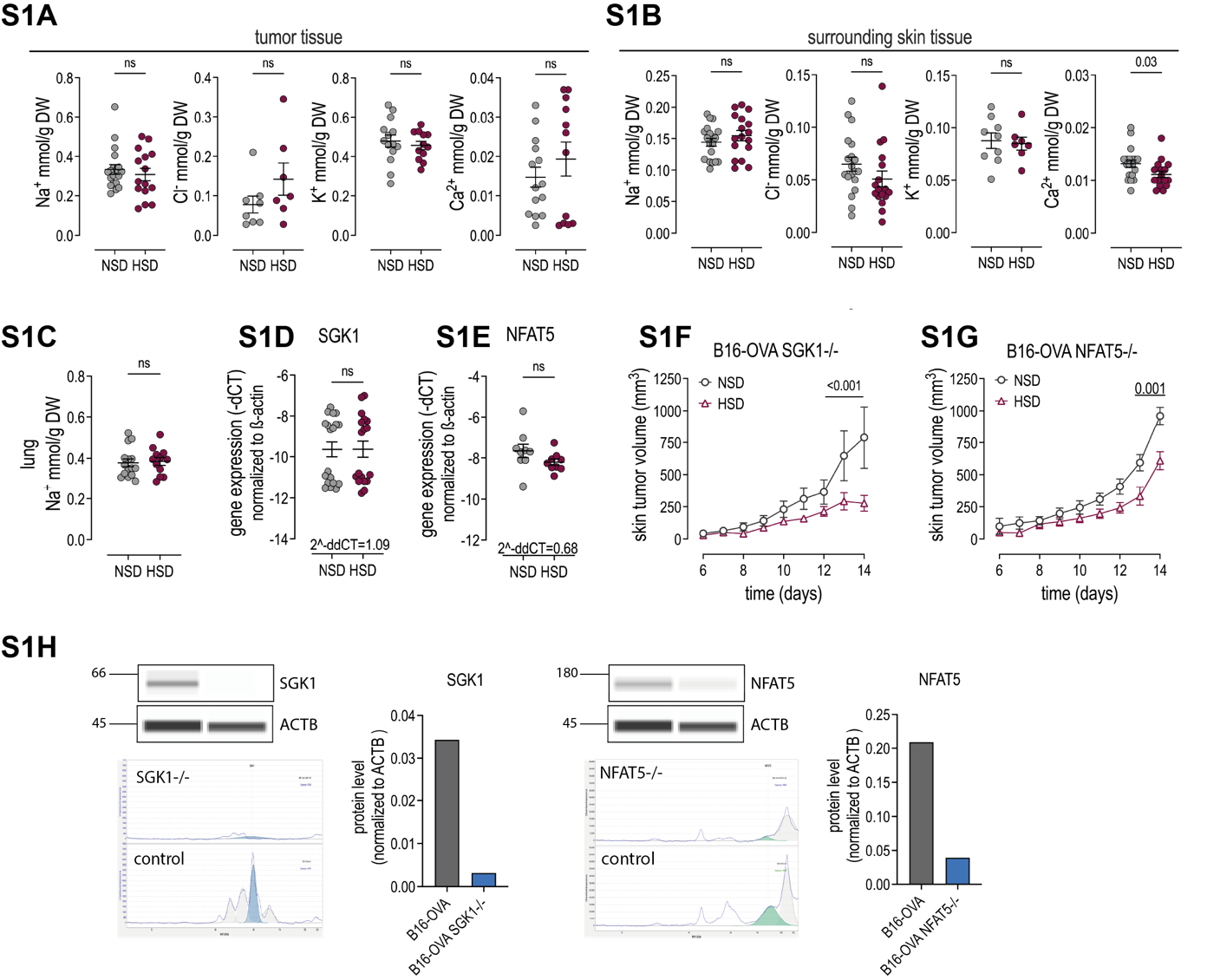
**

**Supplementary figure 1.** (**A**) Ion concentration in *ex vivo* tumor tissue, normalized to its dry weight. (**B**) Ion concentration of skin surrounding the tumor, normalized to its dry weight. (**C**) Sodium concentration in lungs isolated from mice on NSD and HSD. (**D**) Gene expression level of SGK1 in skin tumors *ex vivo*. (**E**) Gene expression level of NFAT5 in skin tumors *ex vivo*. (**F**) Tumor growth (skin) of B16-OVA SGK1^-/-^ cells. (**G**) Tumor growth (skin) of B16-OVA NFAT5^-/-^ cells. (**H**) Western blot (WES) analysis of B16-OVA NFAT5⁻/⁻ and B16-OVA SGK1⁻/⁻ cell lines, accompanied by histograms showing AUC analysis to confirm target protein depletion in the respective cell lines. A-E. n=1-4 independent experiments with 3-10 mice/group each. Linear mixed effect model with treatment (HSD vs. NSD) as fixed factor and experimental replicate as random factor. Data are presented as mean ± SEM. F,G. Tumor volume of B16-OVA SGK1^-/-^ or NFAT5^-/-^ skin melanoma over the course of 14 days. n=2 independent experiments each with 5 mice/group. 2-way-ANOVA, with Sidaks multiple comparison test.

Fig. S2. HSD does not interfere with engraftment of tumor cells

**
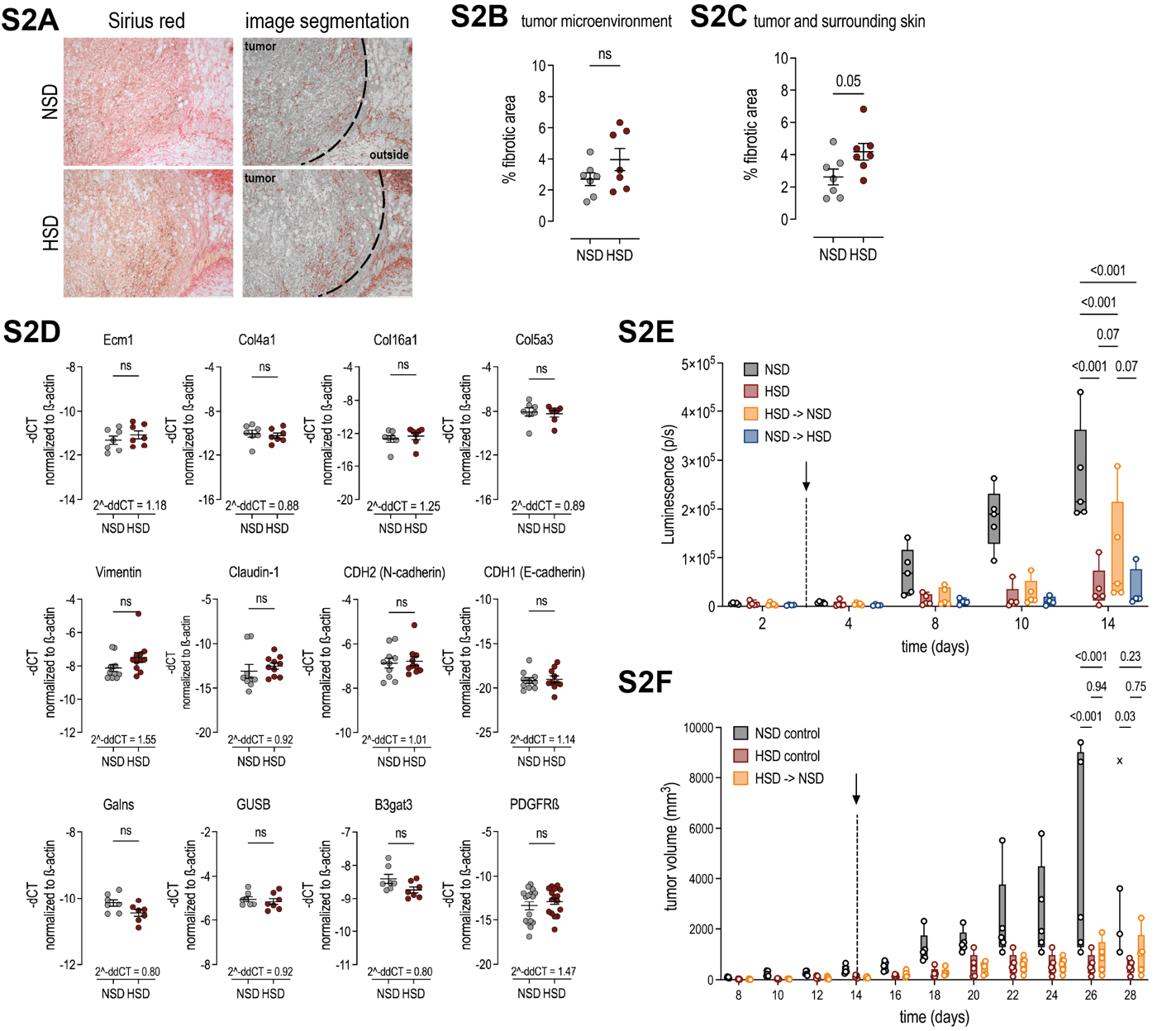
**

**Supplementary figure 2.** (**A**) Representative images of Sirius red staining of skin tumors with surrounding skin (left panel) as well as image segmentation analysis (right panel). (**B**) Fibrotic area of the tumor microenvironment. n=2 independent experiments with 3-4 mice/group each. Linear mixed effect model with treatment (HSD vs. NSD) as fixed factor and experimental replicate as random factor. Data are presented as mean ± SEM. (**C**) Fibrotic area of the skin surrounding the tumor. n=2 independent experiments with 3-4 mice/group each. Linear mixed effect model with treatment (HSD vs. NSD) as fixed factor and experimental replicate as random factor. Data are presented as mean ± SEM. Image segmentation was done with ImageJ. (**D**) Gene expression level of several genes involved in extracellular matrix rearrangement and fibrosis. n=1-2 experiments with 5 mice/group each. Linear mixed effect model with treatment (HSD vs. NSD) as fixed factor and experimental replicate as random factor. Data are presented as mean ± SEM. (**E**) Luminescence, representative of lung melanoma burden, over the course of 14 days. After 3 days, the diet was changed from NSD to HSD or from HSD to NSD. n=1 experiment with 4-5 mice/group each. 2-way-ANOVA, with Sidaks multiple comparison test. (**F**) Tumor volume of B16-OVA skin melanoma over the course of 28 days. n=1 experiment with 5-10 mice/group each. After 14 days, the diet of one group of HSD fed mice was changed to NSD. 2-way-ANOVA, with Sidaks multiple comparison test.

Fig. S3. Respiratory activity of mitochondria during HSD

**
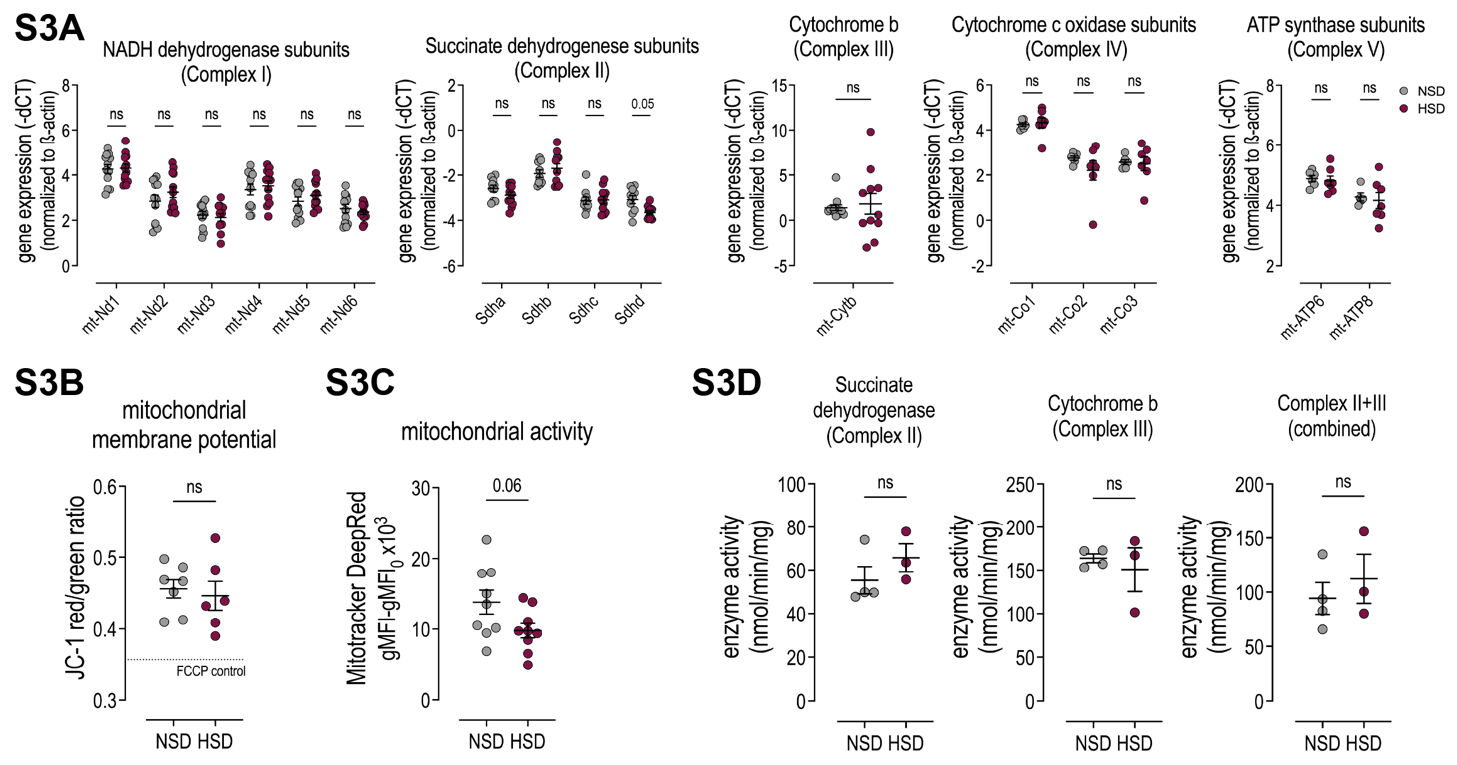
**

**Supplementary figure 3.** (**A**) Gene expression level of proteins (or their subunits) that are part of the mitochondrial electron transport chain. Mitochondria were isolated from tumor cells *ex vivo*. n=1-2 independent experiments with 3-5 mice/group each. 2-way-ANOVA, with Sidaks multiple comparison test. (**B**) JC-1 staining of isolated mitochondria from NSD and HSD tumors, indicative for mitochondrial membrane potential. n=1 experiment with 7 mice/group. Unpaired t-test. Data are presented as mean ± SEM. (**C**) Mitochondrial activity determined by membrane potential dependend MitoTracker DeepRed staining. Measured in tumor cells *ex vivo*. n=1 experiment with 9 mice/group. Unpaired t-test. Data are presented as mean ± SEM. (**D**) Enzyme activity of complex II and complex III of isolated mitochondria (tumor cells *ex vivo*). n=1 experiment with 3-4 mice/group. Unpaired t-test. Data are presented as mean ± SEM.

Fig. S4. Metabolite profile

**
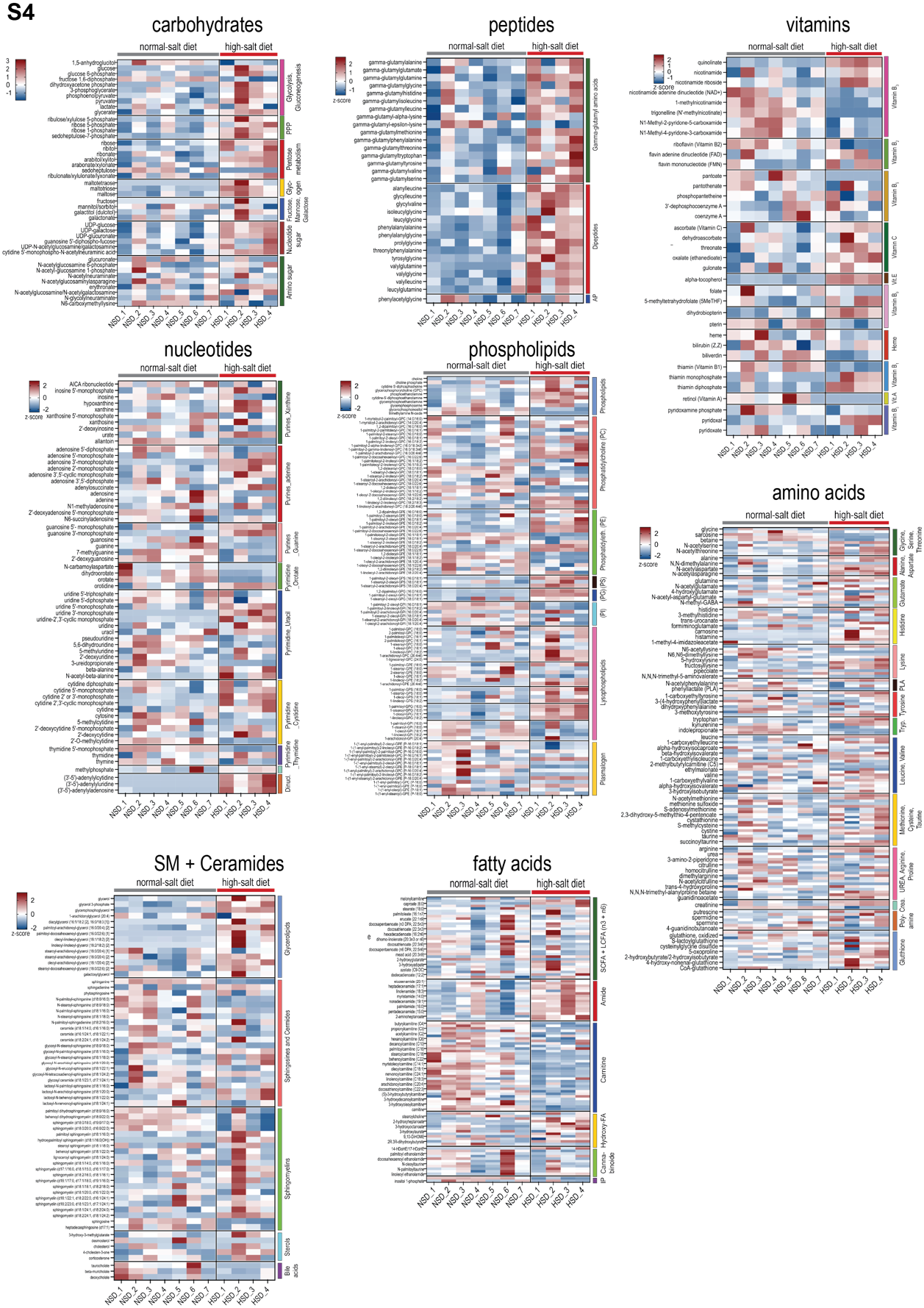
**

**Supplementary figure 4.** Heatmaps (z-score) of metabolites from tumor samples isolated from mice on NSD and HSD. n=1 experiment with 4-7 mice/group.

Fig. S5. Melanogenesis is upregulated independent of a-MSH

**
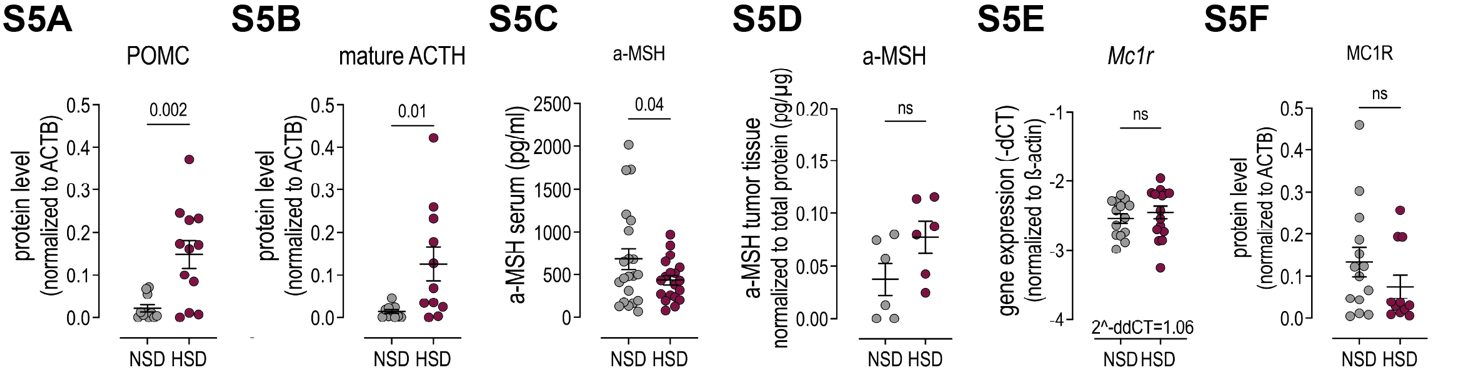
**

**Supplementary figure 5.** (**A**) POMC level in hypothalamus/pituitary glands of tumor-bearing mice on NSD and HSD. n=2 independent experiments with 5-7 mice/group each. (**B**) Mature ACTH level in hypothalamus/pituitary glands of tumor-bearing mice on NSD and HSD. n=2 independent experiments with 5-7 mice/group each. (**C**) a-MSH level in serum of tumor-bearing mice on NSD and HSD. n=5 independent experiments with 3-5 mice/group each. (**D**) a-MSH level in tumor homogenate, normalized to total protein. n=2 independent experiments with 3 mice/group each. (**E**) Gene expression level of *Mc1r* in tumor cells *ex vivo*. n=5 independent experiments with 3-5 mice/group each. (**F**) Protein level of MC1R in tumor cells *ex vivo*. n=4 independent experiments with 3-5 mice/group each. A-F. Linear mixed effect model with treatment (HSD vs. NSD) as fixed factor and experimental replicate as random factor. Data presented as mean ± SEM.

Fig. S6. Simple Western™ (WES) blot


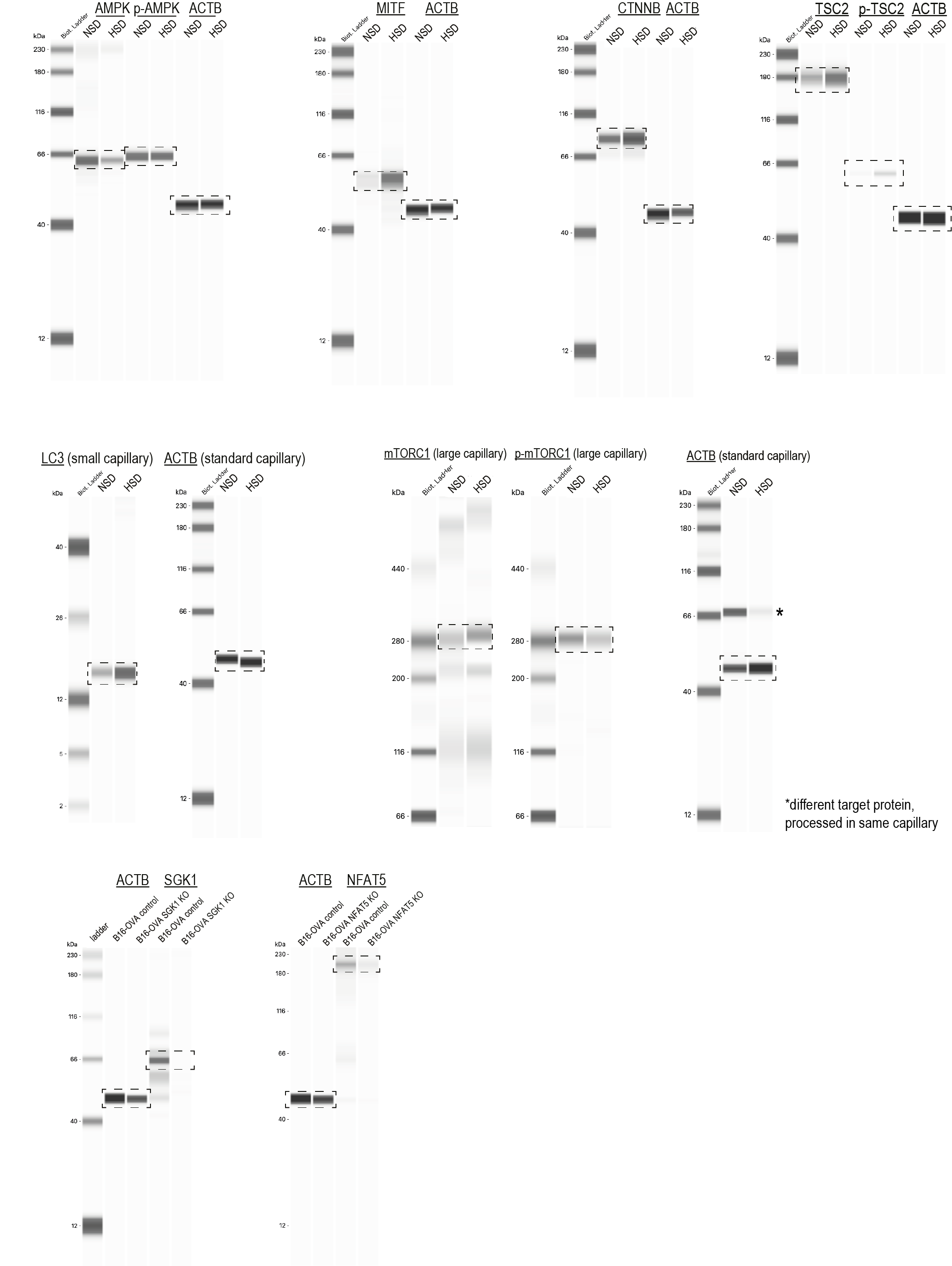


**Supplementary figure 6.** Automated capillary western analysis (Simple Western™) using capillary electrophoresis for protein separation followed by immunoassay-based detection. Full capillary blots of protein samples shown in manuscript. AMPK = 5' adenosine monophosphate-activated protein kinase. MITF = Microphthalmia-associated transcription factor. CTNNB = beta-catenin1, TSC2 = tuberin, LC3 = Microtubule-associated proteins 1A/1B light chain 3B, mTORC1 = mechanistic Target of Rapamycin complex 1, ACTB = beta-actin. SGK1 = serum-glucocorticoid-regulated kinase 1, NFAT5 = tonicity-responsive enhancer binding protein. Dashed squares indicate bands used in main manuscript.

Fig. S7. Immune cells frequencies, gating strategies and isotype controls


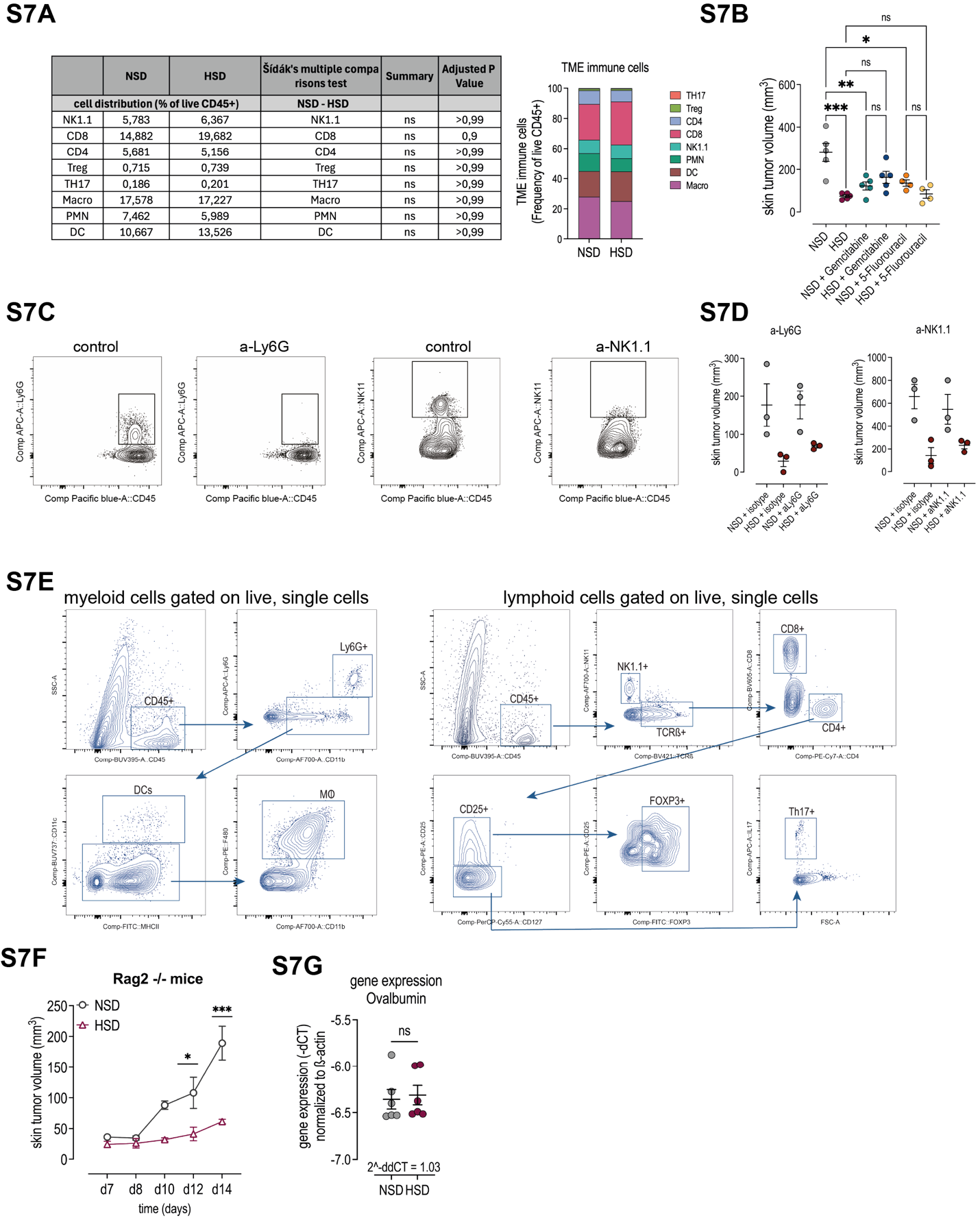


**Supplementary figure 7.** (**A**) Proportions of tumor-associated immune cells corresponding to Figure 2A. (**B**) Skin tumor volume B16-OVA melanoma on day 14 after treatment with gemcitabine and 5-Fluorouracil. n= one experiment with 4-5 mice/group. One-way ANOVA with Sidak’s multiple comparison test. (**C**) Representative flow cytometry plots demonstrating successful depletion in mice treated with anti-Ly6G antibodies (left panel) or anti-NK1.1 antibodies (right panel). (**D**) Skin tumor volume of B16-OVA on day 14, corresponding to Figure 2D, including the respective isotype controls. (**E**) Representative flow cytometry plots illustrating gating strategies used to assess tumor-associated myeloid cells (left) and lymphoid cells (right). (**F**) Growth kinetics of B16-OVA skin tumors in RAG-/- mice corresponding to Figure 2C. (**G**) Gene expression of Ovalbumin in skin tumor tissue on day 14. n= one representative experiment. Unpaired students t-test. Data are shown as mean + SEM.

Fig. S8. Body weight, *bififobacterium* and hippurate level


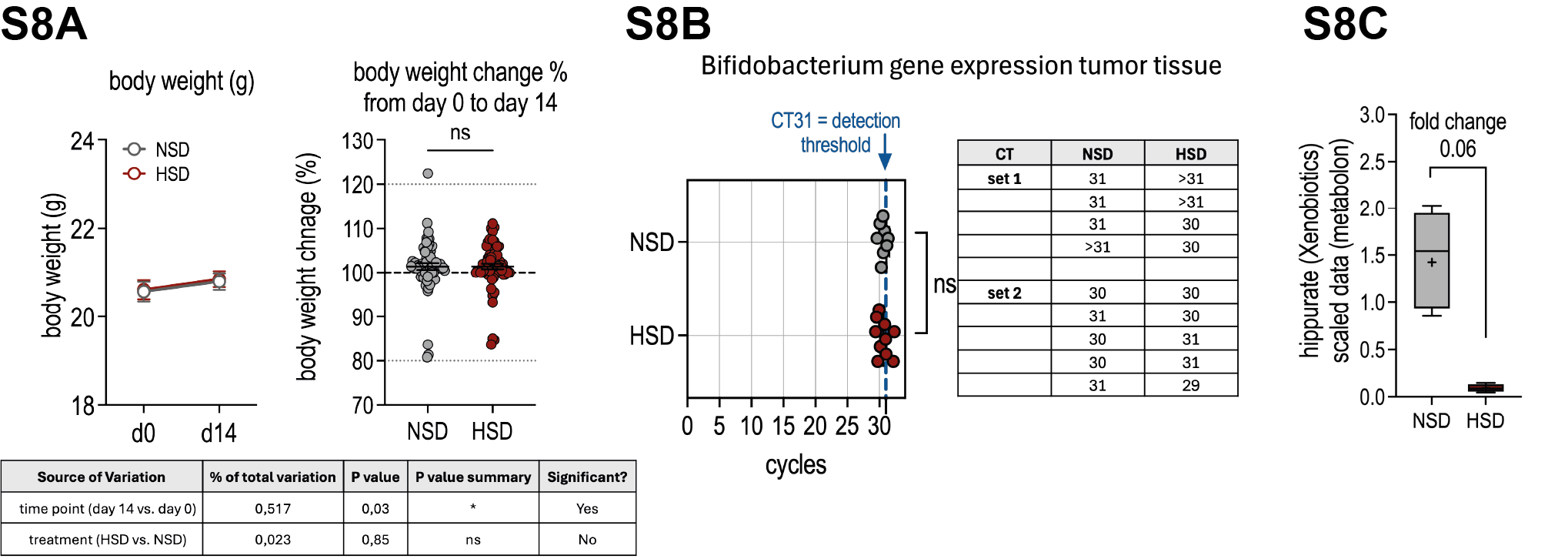


**Supplementary figure 8.** (**A**) Body weight of mice was monitored throughout the experiment (day 0 vs. day 14; left panel) and expressed as the percentage of body weight change for each individual mouse (right panel). Data represent 9 independent experiments with 3–5 mice per group. (**B**) Gene expression levels of Bifidobacterium were assessed in B16-OVA tumor tissue on day 14 from mice on NSD or HSD. n= one experiment with 4–5 mice per group. CT values were at or below the detection limit, indicating the absence of Bifidobacterium in the tumor tissue. (**C**) Hippurate level in from tumor samples isolated from mice on NSD and HSD. n=1 experiment with 4-7 mice/group (determined by Metabolon)

Fig. S9. Effect of sodium in-vitro


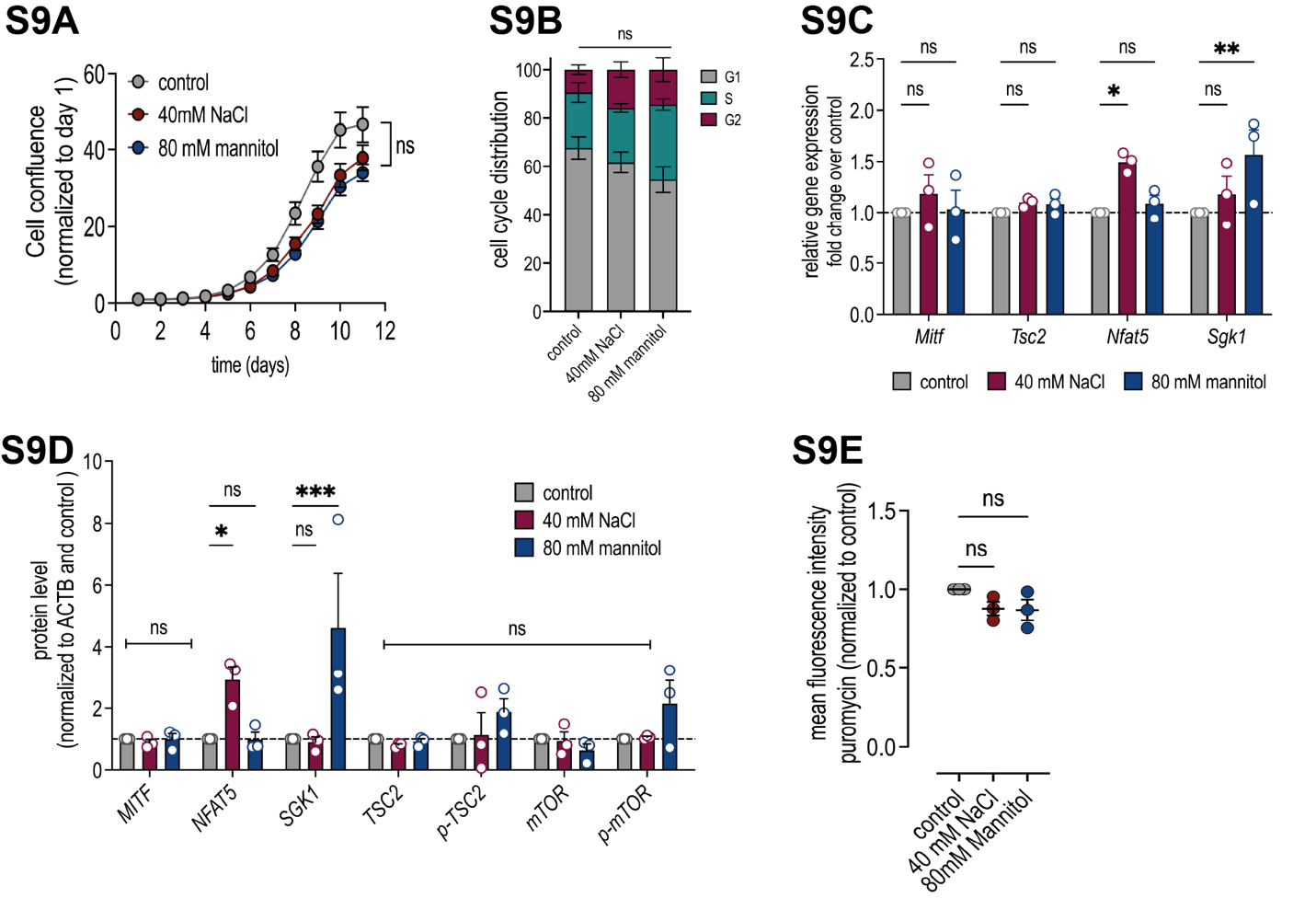


**Supplementary figure 9.** (**A**) Cell confluence analysis over the course of 11 days (until 100% confluence was reached) of B16-OVA cells treated with 40 mM NaCl or 80 mM mannitol. Automated measurement was done a using the Tecan Spark Cell confluence module®. (**B**) Cell cycle analysis of B16-OVA cells treated with 40 mM NaCl or 80 mM mannitol for 14 days. (**C**) Gene expression of *Mitf* (indicative for melanogenesis), *Tsc2* (indicative for metabolism), *Nfat5* and *Sgk1*, indicating sodium or osmotic pressure response. (**D**) Protein level of MITF, NFAT5, SGK1, TSC2, p-TSC2, mTOR and p-mTOR in B16-OVA cells treated with 40 mM NaCl or 80 mM mannitol for 14 days. (**E**) Mean fluorescence puromycin, indicative of protein synthesis in B16-OVA cells treated with 40 mM NaCl or 80 mM mannitol for 14 days. N= 3 independent experiments. 2-way-ANOVA, with Sidaks multiple comparison test.

Fig. S10. Influence of HSD on B16V melanoma


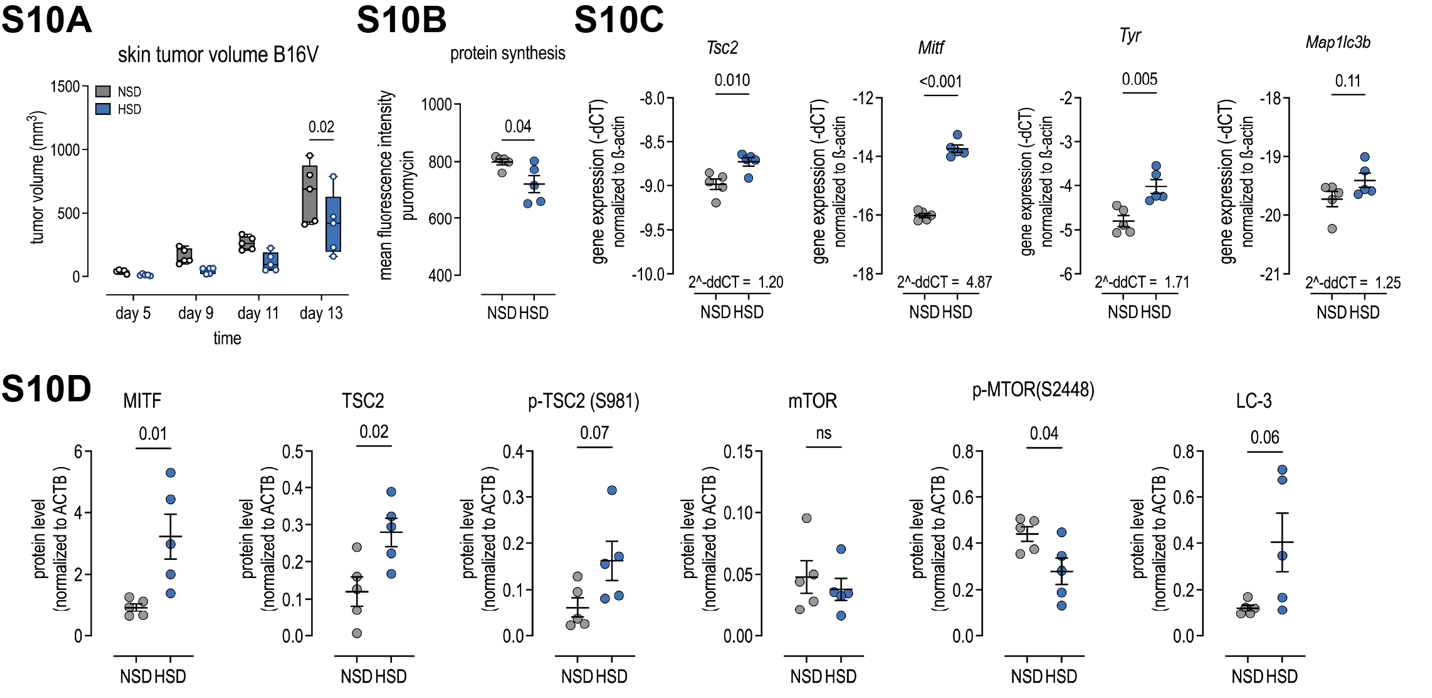


Supplementary figure 10. (A) Skin tumor volume of B16V tumors injected s.c. in mice fed NSD or HSD for 7 days before tumor injection. Tumor growth was determined for 14 days, while maintaining the respective diets. (B) Mean fluorescence intensity of incorporated puromycin (ex vivo), indicative for protein synthesis on B16V cells isolated on day 14. (C) Gene expression level of Tsc2, Mitf, tyrosinase and Map1lc3b in B16V tumors isolated from mice on NSD or HSD on day 14. (D) Protein level of MITF, TSC2, p-TSC2 (S981), mTOR, p-mTOR (S2448) and LC-3 in B16V tumors isolated on day 14 from mice fed NSD or HSD.
