# Supplementary figures and images for: High-salt diet induces immune-independent re-differentiation, metabolic shut down and cell cycle arrest of melanoma
Source: Cell Death Dis. 2025 Dec 20;17(1):102. doi: 10.1038/s41419-025-08329-x (PMC12847697; doi:10.1038/s41419-025-08329-x)

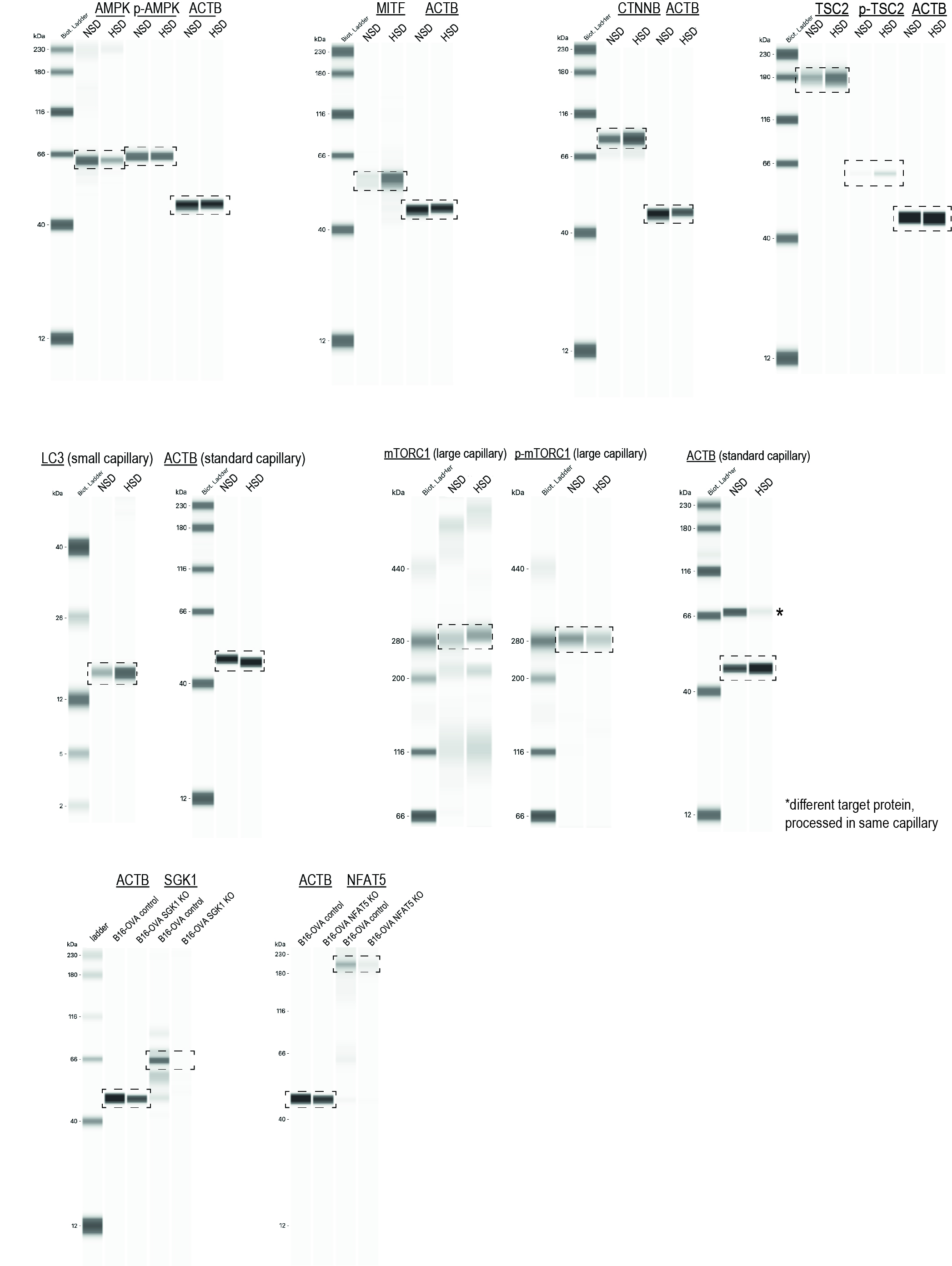

Supplement: Supplementary file 2 — Original Data_western blot [file 41419_2025_8329_MOESM2_ESM.jpg]
